# The role of vascular risk factors in white matter tract microstructure: a multi‐cohort study in older adults

**DOI:** 10.1002/alz.71698

**Published:** 2026-07-31

**Authors:** James D. LeFevre, Yukti Vyas, Aditi Sathe, Niranjana Shashikumar, Kimberly R. Pechman, Yisu Yang, Alaina Durant, Praitayini Kanakaraj, Michael E. Kim, Chenyu Gao, Nancy R. Newlin, Karthik Ramadass, Nazirah Mohd Khairi, Zhiyuan Li, Tianyuan Yao, Shannon L. Risacher, Panpan Zhang, Kurt G. Schilling, Annie J. Lee, Adam M. Brickman, Richard Mayeux, Jesse Mez, for the Alzheimer's Disease Neuroimaging Initiative (ADNI), The Alzheimer's Disease Sequencing Project (ADSP)The ADSP Phenotype Harmonization Consortium (ADSP‐PHC), Walter Kukull, Sarah A. Biber, Bennett A. Landman, Barbara B. Bendlin, Sterling C. Johnson, Julie Schneider, Lisa L. Barnes, David A. Bennett, Andrew J. Saykin, Michael L. Cuccaro, Timothy J. Hohman, Angela L. Jefferson, Derek B. Archer

**Affiliations:** ^1^ Vanderbilt Memory and Alzheimer's Center Vanderbilt University School of Medicine Nashville Tennessee USA; ^2^ Vanderbilt Alzheimer's Disease Research Center Vanderbilt University Nashville Tennessee USA; ^3^ Department of Computer Science Vanderbilt University Nashville Tennessee USA; ^4^ Department of Electrical and Computer Engineering Vanderbilt University Nashville Tennessee USA; ^5^ Digital Informatics & Technology Solutions Memorial Sloan Kettering Cancer Center New York New York USA; ^6^ Department of Computer Science Park University Parkville Missouri USA; ^7^ Department of Radiology and Imaging Sciences Indiana University School of Medicine Indianapolis Indiana USA; ^8^ Indiana Alzheimer's Disease Research Center Indiana University School of Medicine Indianapolis Indiana USA; ^9^ Department of Biostatistics Vanderbilt University Medical Center Nashville Tennessee USA; ^10^ Department of Radiology & Radiological Sciences Vanderbilt University Medical Center Nashville Tennessee USA; ^11^ Vanderbilt University Institute of Imaging Science Vanderbilt University Medical Center Nashville Tennessee USA; ^12^ Department of Neurology Columbia University Irving Medical Center New York New York USA; ^13^ Taub Institute for Research on Alzheimer's Disease and the Aging Brain Columbia University New York New York USA; ^14^ The Gertrude H. Sergievsky Center, College of Physicians and Surgeons, Columbia University New York New York USA; ^15^ Department of Neurology, Vagelos College of Physicians and Surgeons, Columbia University, and the New York Presbyterian Hospital New York New York USA; ^16^ The Institute for Genomic Medicine Columbia University Medical Center New York New York USA; ^17^ Department of Neurology Boston University Chobanian & Avedisian School of Medicine Boston Massachusetts USA; ^18^ Boston University Alzheimer's Disease Research Center Boston University Chobanian & Avedisian School of Medicine Boston Massachusetts USA; ^19^ Department of Neurology Washington University at St. Louis St. Louis Missouri USA; ^20^ Vanderbilt Brain Institute Vanderbilt University Medical Center Nashville Tennessee USA; ^21^ Department of Biomedical Engineering Vanderbilt University Nashville Tennessee USA; ^22^ Wisconsin Alzheimer's Disease Research Center School of Medicine and Public Health, University of Wisconsin Madison Wisconsin USA; ^23^ Wisconsin Alzheimer's Institute School of Medicine and Public Health, University of Wisconsin Madison Wisconsin USA; ^24^ Rush Alzheimer's Disease Center Rush University Medical Center Chicago Illinois USA; ^25^ The John P. Hussman Institute for Human Genomics University of Miami Miami Florida USA; ^26^ Dr. John T. MacDonald Foundation, Department of Human Genetics University of Miami Miami Florida USA; ^27^ Vanderbilt Genetics Institute Vanderbilt University Medical Center Nashville Tennessee USA; ^28^ Department of Neurology Vanderbilt University Medical Center Nashville Tennessee USA; ^29^ Department of Medicine Vanderbilt University Medical Center Nashville Tennessee USA

**Keywords:** aging, diffusion tensor imaging, tractography, vascular risk factors, white matter microstructure

## Abstract

**INTRODUCTION:**

Vascular risk factors (VRFs) contribute to white matter microstructural degeneration, but their tract‐specific contributions are unclear. We assessed the independent associations of four major VRFs with white matter microstructure in a large, multi‐cohort study.

**METHODS:**

Diffusion magnetic resonance imaging data from five harmonized cohorts were free water (FW) corrected (*n* = 2961, 73.00 ± 9.27 years, 59.34% female). We associated body mass index (BMI) and presence of hypertension, diabetes, and heart disease with FW and FW‐corrected diffusion metrics (fractional anisotropy, mean diffusivity, axial diffusivity, radial diffusivity) in 48 tracts.

**RESULTS:**

Hypertension and heart disease showed the most robust and widespread independent associations with white matter microstructure. In contrast, diabetes associations were attenuated in sensitivity analyses, while BMI associations were inconsistent across metrics.

**DISCUSSION:**

Hypertension and heart disease are associated most strongly with white matter microstructure, suggesting that tighter management of these conditions may more reliably yield benefits in preserving white matter health.

## Highlights

- Harmonized MRI data from five cohorts (*n* = 2961) assessed vascular risk factors.
- Hypertension and heart disease showed widespread white matter tract associations.
- Hypertension contributions were strongest in the fornix and pre‐SMA TC tract.
- Heart disease contributions were strongest in the cingulum and ILF.
- Diabetes and BMI showed fewer associations with white matter tracts.

## 1 BACKGROUND

Neurological and cardiovascular diseases are major contributors to mortality and disability worldwide,^1^, ^2^ with their burden expected to grow due to population growth and increasing life expectancy.^3^, ^4^ Consequently, understanding the pathophysiology and relations between cerebral and cardiovascular conditions is imperative. Understanding how vascular risk factors (VRFs) affect cognition could help develop therapeutic strategies to mitigate this burden.

VRFs exert deleterious effects on vasculature throughout the body, including the brain.^5^, ^6^ These effects are associated with cognitive decline and changes in white matter microstructure,^6^, ^7^, ^8^, ^9^ but tract‐specific changes have been less studied. Myelinated white matter tracts enable efficient neural communication and cognitive processing. The integrity of these tracts can be assessed with diffusion tensor imaging (DTI), which measures the diffusion of water molecules to quantify key characteristics of white matter microstructure.^10^ Free water (FW) fractional anisotropy (FA), mean diffusivity (MD), axial diffusivity (AxD), and radial diffusivity (RD) are conventional DTI metrics that measure different aspects of white matter microstructure. FW quantifies the volume fraction of water molecules exhibiting unrestricted diffusion in the extracellular space that is not constrained by cell membranes. FA measures the degree of directionality of water diffusion, with higher FA indicating higher directional diffusion (e.g., along intact white matter fibers) and lower FA indicating reduced directionality, suggesting that the white matter fibers are less organized or have been disrupted (e.g., in damaged tissue where diffusion occurs equally in all directions). In contrast, MD represents the average rate of water diffusion within the tissue, AxD measures the rate of diffusion parallel to the white matter fiber axons, and RD quantifies the rate of diffusion perpendicular to the white matter fiber axons. Conventional single‐tensor metrics are limited by partial volume effects,^10^ which bi‐tensor models address by estimating and correcting for voxel FW content.^11^ FW correction more accurately quantifies microstructure by capturing FW‐corrected FA (FA_FWcorr_), MD (MD_FWcorr_), AxD (AxD_FWcorr_), and RD (RD_FWcorr_). Prior work showed that FW‐corrected white matter metrics were abnormal along the Alzheimer's disease (AD) continuum^12^ and associated with cognitive decline^13^ and memory decline independently of gray matter atrophy.^14^ The combination of FW‐corrected DTI metrics and large‐scale harmonization of well‐established cohorts offers substantial advantages for examining the relationships between VRFs and white matter microstructure.

While numerous studies have demonstrated associations between VRFs and white matter microstructure, their independent and differential contributions remain to be quantified, particularly as these factors frequently co‐occur in aging populations. Here, we determined the relative associations of VRFs with white matter microstructure across 48 tracts in a large, multi‐cohort dataset, utilizing multiple DTI metrics to capture distinct microstructural alterations. These risk factors operate through overlapping and distinct mechanisms. Hypertension's primary mechanism exerts chronic mechanical stress on vessel walls promoting endothelial dysfunction, arterial stiffening, and arteriolosclerosis,^15^ whereas heart disease often results in systemic hypoperfusion and reduction in cerebral blood flow.^16^ Conversely, diabetes and body mass index (BMI) promote metabolic dysregulation and inflammation.^17^, ^18^ Given these distinct pathophysiological pathways, we hypothesized that individual VRFs would exhibit differential associations with white matter microstructure, reflecting their unique underlying vascular mechanisms.

**RESEARCH IN CONTEXT**

1. **Systematic review**: The authors reviewed articles using PubMed and Google Scholar linking VRFs to white matter microstructure. While prior studies associated aggregate vascular risk with white matter degeneration, few used large‐scale, harmonized multi‐cohort data to determine the unique associations of hypertension, diabetes, heart disease, and BMI with specific white matter tracts. Furthermore, previous work often lacked FW‐corrected metrics, which are crucial for distinguishing microstructural changes from FW contamination in aging populations.
2. **Interpretation**: Hypertension and heart disease exhibited the most robust and widespread independent associations with white matter microstructure, whereas diabetes and BMI showed limited contributions. These results suggest that hypertension and heart disease are the primary vascular drivers of white matter injury in older adults.
3. **Future directions**: Future research needs to investigate how the severity and duration of VRFs differentially affect white matter tracts and the unique biological mechanisms underlying their associations.

## 2 METHODS

### 2.1 Study cohort

The Alzheimer's Disease Sequencing Project Phenotype Harmonization Consortium (ADSP‐PHC) began in 2021 to provide large‐scale harmonization of ADSP cohorts, spanning markers of cognition, neuroimaging, VRFs, fluid biomarkers, and neuropathology. The cohorts in ADSP‐PHC that were included in this study were Alzheimer's Disease Neuroimaging Initiative (ADNI), the National Alzheimer's Coordinating Center (NACC) data set, the Religious Orders Study/Rush Memory and Aging Project/Minority Aging Research Study (ROS/MAP/MARS), and the Wisconsin Registry for Alzheimer's Prevention (WRAP). The Vanderbilt Memory and Aging Project (VMAP) was also included.

Data used in the preparation of this article were obtained from the ADNI database (adni.loni.usc.edu). The ADNI was launched in 2003 as a public‐private partnership, led by Principal Investigator Michael W. Weiner, MD. The primary goal of ADNI has been to test whether serial magnetic resonance imaging (MRI), positron emission tomography, other biological markers, and clinical and neuropsychological assessment can be combined to measure the progression of mild cognitive impairment and early AD.^19^ Three ADNI phases (ADNI‐GO, ADNI 2, and ADNI 3) were included in this study. NACC maintains a large, publicly available database of standardized clinical and neuropathological data from National Institute on Aging‐funded Alzheimer's Disease Research Centers.^20^ ROS/MAP/MARS are longitudinal, epidemiologic clinical–pathological cohort studies that were designed to characterize common chronic conditions of aging and the neuropathological basis of cognitive impairment. ROS began in 1994 and enrolled older Catholic priests, nuns, and brothers across the United States.^21^ MAP began in 1997 and enrolled older men and women in the Chicagoland area.^21^ MARS began in 2004 and enrolled older adults who self‐identified as Black in the Chicagoland area.^22^ These cohorts share a common data core and imaging pipeline, facilitating efficient data merging.^23^ VMAP is a longitudinal observational study that began in 2012 to investigate the relationship between vascular health and brain aging.^24^ The WRAP, which began in 2001, is a longitudinal, observational study on midlife adults who do not have dementia and is enriched with persons with a parental history of AD.^25^ In addition to each cohort's own inclusion/exclusion criteria, covariates and VRFs used in this study were required for inclusion. Included participants required diffusion MRI (dMRI), demographic, and clinical data, were ≥50 years old, and passed neuroimaging quality control. Across all cohorts, written informed consent was provided by participants, and research followed approved Institutional Review Board (IRB) protocols. Secondary analysis of these data was approved by the Vanderbilt University IRB.

Table 1 provides an overview of the ADNI, NACC, ROS/MAP/MARS, VMAP, and WRAP sample sizes, demographic information, and health characteristics. All participants had a primary diagnosis of normal cognition, mild cognitive impairment, or AD dementia. Participants were not excluded based on the presence of cerebral small‐vessel disease, and individuals with other neurodegenerative conditions were not included.

**TABLE 1:** Participant characteristics.

Measure	Cohort					
	ADNI	NACC	ROS/MAP/MARS	VMAP	WRAP	Total
**Cohort characteristics**						
Total number of participants	1018	541	811	526	68	2964
**Demographic characteristics**						
Age (years)	74.24 ± 7.97	75.34 ± 9.28	82.90 ± 7.40	70.86 ± 9.13	64.69 ± 5.98	75.99 ± 9.48
Absolute interval between DTI and VRF assessment (years)	0.82 ± 1.18	2.34 ± 1.63	2.41 ± 1.35	0.34 ± 0.26	2.57 ± 1.73	1.49 ± 1.52
Sex (% female)	51.67	57.49	75.71	49.62	69.12	59.35
Education (years)	16.33 ± 2.47	14.16	15.89 ± 3.24	16.08 ± 2.49	16.37 ± 1.94	15.77 ± 3.10
Race (% non‐Hispanic White)	77.11	67.65	73.49	85.55	97.06	76.35
Cognitive status at baseline (% cognitively unimpaired)	48.13	43.99	76.08	76.81	97.06	61.23
Systolic blood pressure (mmHg)	133.53 ± 17.75	137.58 ± 19.35	129.36 ± 19.44	135.25 ± 17.42	129.84 ± 19.02	133.32 ± 18.71
**Vascular risk factors**						
Hypertension (%)	46.66	88.72	92.36	63.45	35.29	69.74
Diabetes (%)	63.46	26.84	23.55	15.78	4.41	36.40
Heart disease (%)	61.69	25.88	24.04	5.51	32.35	34.21
Body mass index (kg/m^2^)	27.46 ± 5.34	27.10 ± 5.12	27.12 ± 5.40	27.68 ± 4.87	28.75 ± 6.25	27.37 ± 5.26

*Note*. Data reflect the sample included in the 5‐year restricted primary analysis. Values denoted as mean ± standard deviation or frequency.

Abbreviations: ADNI, Alzheimer's Disease Neuroimaging Initiative; MAP, Rush Memory and Aging Project; MARS, Minority Aging Research Study; NACC, National Alzheimer's Coordinating Center; ROS, Religious Orders Study; VMAP, Vanderbilt Memory and Aging Project; WRAP, Wisconsin Registry for Alzheimer's Prevention.

### 2.2 Diffusion MRI data acquisition and preprocessing

**FIGURE 1:**
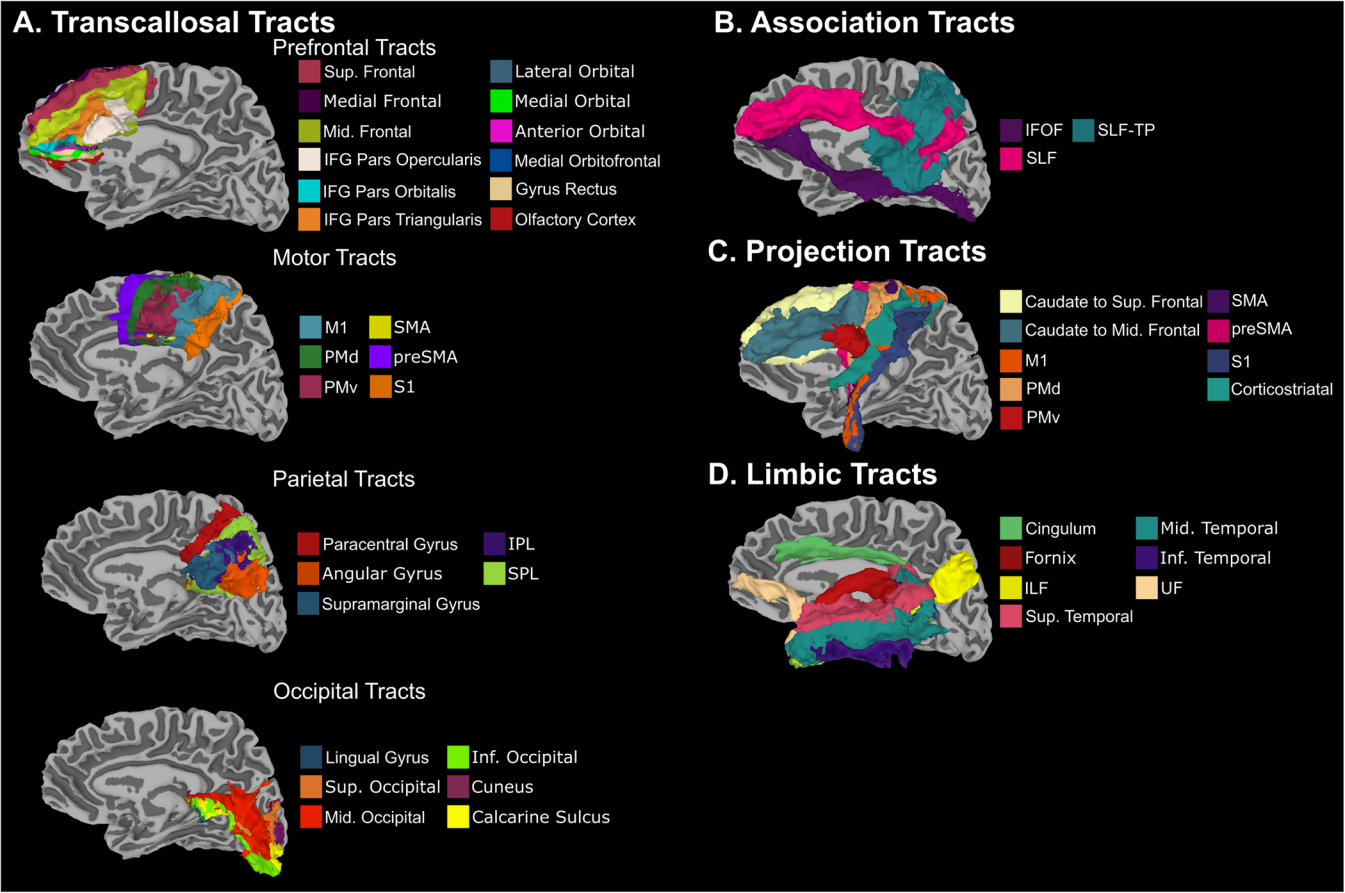
White matter tractography templates. Forty‐eight white matter tractography templates were used in this study and can be grouped into TC (A), association (B), projection (C), and limbic tracts (D). IFG, inferior frontal gyrus; IFOF, inferior fronto‐occipital fasciculus; ILF, inferior longitudinal fasciculus; IPL, inferior parietal lobe; M1, primary motor cortex; PMd, dorsal premotor cortex; PMv, ventral premotor; S1, primary somatosensory cortex; SLF, superior longitudinal fasciculus; SLF‐TP, temporoparietal superior longitudinal fasciculus; SMA, supplementary motor area; SPL, superior parietal lobe; TC, transcallosal; UF, uncinate fasciculus.

Image processing and quantification followed previously described protocols.^13^, ^26^, ^27^ Briefly, dMRI data were preprocessed using the *PreQual* automated pipeline,^28^, ^29^ followed by FW‐corrected metric calculation^11^ and non‐linear registration to the FMRIB58_FA atlas.^30^ We quantified FW and FW‐corrected metrics (FA_FWcorr_, MD_FWcorr_, AxD_FWcorr_, RD_FWcorr_) across 48 standard white matter tract templates^31^ (Figure 1). Data were harmonized using the *Longitudinal ComBat* technique in R, controlling for all *site* × *scanner* × *protocol* combinations.^32^ Harmonization covariates included sex, diagnosis, mean‐centered age, mean‐centered age squared, and age interactions with converter status (non‑converter and converter). Non‐converters were cognitively unimpaired at all dMRI timepoints, and converters had a non‑cognitively unimpaired diagnosis (mild cognitive impairment or dementia) at any timepoint. A detailed summary of dMRI acquisition parameters for each cohort is provided in Table S1.

### 2.3 Assessment of VRFs

To assess vascular risk, history of hypertension, diabetes, heart disease, and BMI were included in this study. VRFs were primarily derived from self‐reported data across most cohorts; however, in VMAP and the ROS/MAP/MARS cohorts, more rigorous clinical criteria were applied to determine the presence of hypertension and diabetes. In VMAP, the two blood pressure measurements were taken during the same echocardiogram visit, and the mean of the two readings was used. Hypertension was defined as systolic blood pressure ≥ 140 mmHg or diastolic blood pressure ≥ 90 mmHg, or current use of antihypertensive medication. The presence of diabetes was determined by hemoglobin A1c ≥ 6.5%, fasting blood glucose ≥ 126 mg/dL, or oral hypoglycemic medication or insulin use. Similarly, in the ROS/MAP/MARS cohorts, diabetes was identified through a combination of self‐reported medical history, laboratory data including hemoglobin A1c, and a visual inspection of all medication receptacles. History of heart disease was determined by a self‐reported history of angina, coronary artery disease, coronary artery bypass surgery, myocardial infarction, heart failure, or angioplasty. History of hypertension, diabetes, and heart disease was reported as binary (yes or no). Across all cohorts BMI was calculated from height and weight measured directly during the physical examination and was reported on a continuous scale of kg/m^2^.

### 2.4 Covariates

Age, sex, education, self‐reported race/ethnicity, and cognitive status were included as covariates. Age and education were used on a continuous scale in years, while sex (male or female), race/ethnicity (non‐Hispanic white, non‐Hispanic black, other), and cognitive status (normal, mild cognitive impairment, and dementia) were used as categorical variables. Assessments of covariates have been described in greater detail in the respective studies.^20^, ^21^, ^22^, ^24^, ^25^, ^33^

### 2.5 Statistical analyses

To investigate the relative associations of VRFs with white matter microstructure, we conducted a comprehensive series of cross‐sectional analyses using multiple linear regression models. For each participant, VRF data were linked to a DTI scan. We implemented a temporal matching procedure, given that VRF and DTI assessments were not always performed concurrently. We first identified any VRF assessment that occurred within a 5‐year interval of a DTI scan. The 5‑year window refers to the timing of the VRF assessment relative to the DTI scan, not the duration of the condition. Assessments capturing lifetime history were included as long as the assessment date was within 5 years of the scan. If multiple VRF assessments occurred within this window, the assessment closest to the scan was selected. For each participant, only the earliest DTI scan and its corresponding VRF data were included in the analysis.

Our study included baseline FW, FA_FWcorr_, MD_FWcorr_, AxD_FWcorr_, and RD_FWcorr_ across all 48 white matter tracts. Because all four VRFs were entered as predictors in each multiple linear regression model, we tested for multicollinearity among them by calculating variance inflation factors (VIFs). No problematic collinearity was detected (all VIFs < 5).

To assess whether the VRFs related to differences in FW, FA_FWcorr_, MD_FWcorr_, AxD_FWcorr_, and RD_FWcorr_ in each of the 48 white matter tracts, 240 primary multiple linear regression models were created. Each VRF (presence of hypertension, diabetes, and heart disease as binary variables and BMI as a continuous variable) and covariate was included as an independent variable, while FW, FA_FWcorr_, MD_FWcorr_, AxD_FWcorr_, or RD_FWcorr_ served as a dependent variable across the 48 white matter tracts. Covariates included age, sex, education, race/ethnicity, cognitive status, and the time interval (in years) between the DTI and VRF assessments.

To reveal the unique variance explained by each VRF, five reduced models were derived from each primary model. The reduced models omitted one VRF to calculate its partial *R* ^2^ value, the variable's relative association with differences in FW, FA_FWcorr_, MD_FWcorr_, AxD_FWcorr_, or RD_FWcorr_ of the white matter tract beyond the VRFs and covariates. F‐tests were conducted to compare primary versus reduced models to evaluate the independent associations of each VRF.

To ensure the robustness of our findings, we conducted a sensitivity analysis focusing on the influence of outliers and their potential impact on the regression models. Standardized residuals were calculated for each primary model, and residuals exceeding 5 standard deviations from the mean were flagged as potential outliers. Model outputs, histograms, and scatterplots of the residuals with and without outliers were examined to assess data skewness and the distribution of residuals. The exclusion of outliers had minimal influence on the output of the analyses, and therefore, all data points were included in the study. Additional sensitivity analyses were conducted to determine whether to include *APOE* ɛ4 as a covariate. Including *APOE* ɛ4 as a covariate also had minimal impact on the analytical output. Consequently, *APOE* ɛ4 was excluded from the analysis to increase sample size (227 participants had missing *APOE* ɛ4 data). To confirm the robustness of our primary findings and ensure the observed associations were not an artifact of the wider 5‐year temporal window, secondary analyses were repeated on a more temporally restricted sample, including only participants whose DTI and VRF assessments were conducted within 1 year. Significance was set a priori at *p* < 0.05. To control for multiple comparisons across the 48 white matter tracts, the Benjamini‐Hochberg procedure was applied separately for each VRF within each DTI metric. Analyses were conducted with R version 4.4.2.^34^

## 3 RESULTS

### 3.1 VRFs and FW

**FIGURE 2:**
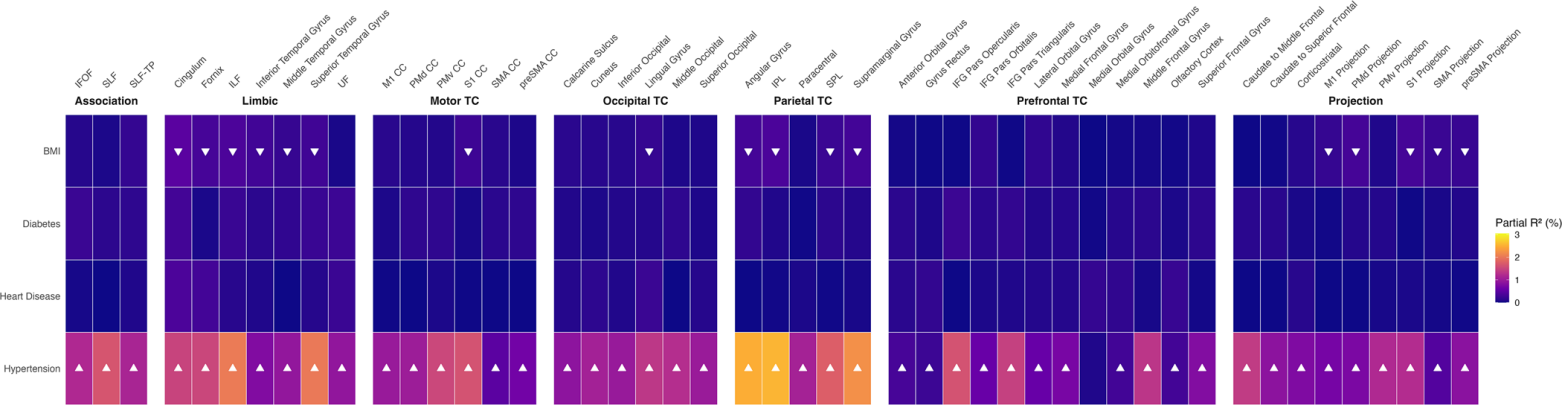
Associations between VRFs and FW. Reduced models of the primary regression models were used to quantify the relative contributions of VRFs to alterations in FW across the 48 white matter tracts. The heatmap illustrates partial *R* ^2^ values, highlighting the relative contributions to FW, from the 5‐year restricted primary analysis. Arrows indicate direction of association, with upward‐pointing arrows indicating a positive beta and downward‐pointing arrows indicating a negative beta. *Tracts surviving correction for multiple comparisons. CC, corpus callosum; FW, free water; IFG, inferior frontal gyrus; IFOF, inferior fronto‐occipital fasciculus; ILF, inferior longitudinal fasciculus; IPL, inferior parietal lobe; M1, primary motor cortex; PMd, dorsal premotor cortex; PMv, ventral premotor; S1, primary somatosensory cortex; SLF, superior longitudinal fasciculus; SLF‐TP, temporoparietal superior longitudinal fasciculus; SMA, supplementary motor area; SPL, superior parietal lobe; TC, transcallosal; UF, uncinate fasciculus; VRF, vascular risk factor.

Associations between VRFs and FW were observed within the 48 white matter tracts (Figure 2). In the primary analyses, hypertension showed widespread associations within 47 of 48 tracts with hypertension associated with higher FW. The strongest effects were observed in the transcallosal (TC) inferior parietal lobe (IPL) (partial *R* ^2^ = 2.5%, *p*_FDR_ < 0.001), angular gyrus (partial *R* ^2^ = 2.4%, *p_FDR_ *< 0.001), and supramarginal gyrus (partial *R* ^2^ = 2.3%, *p_FDR_ *< 0.001) tracts. BMI also showed widespread associations in 17 tracts, with higher BMI associated with higher FW, led by the cingulum (partial *R* ^2^ = 0.52%, *p_FDR_* = 0.0046), inferior longitudinal fasciculus (ILF) (partial *R* ^2^ = 0.41%, *p_FDR_* = 0.013), and fornix (partial *R* ^2^ = 0.37%, *p_FDR_* = 0.013). In contrast, diabetes and heart disease were not associated with FW in any white matter tract after false discovery rate (FDR) adjustment. Refer to Figure S1 for a forest plot and Table S2 for the strongest statistical results.

Secondary 1‐year restricted analyses confirmed hypertension's robust effect across 47 tracts. Conversely, fewer associations for BMI reached statistical significance in the restricted sample, decreasing from 16 to only four tracts. Associations for heart disease emerged in six tracts while diabetes remained non‐significant in the 1‐year restricted analysis. See Table S3 for participant characteristics, Figure S2 for a partial *R* ^2^ heatmap, and Figure S3 for a forest plot from the 1‐year restricted analysis.

### 3.2 VRFs and FA_FWcorr_

**FIGURE 3:**
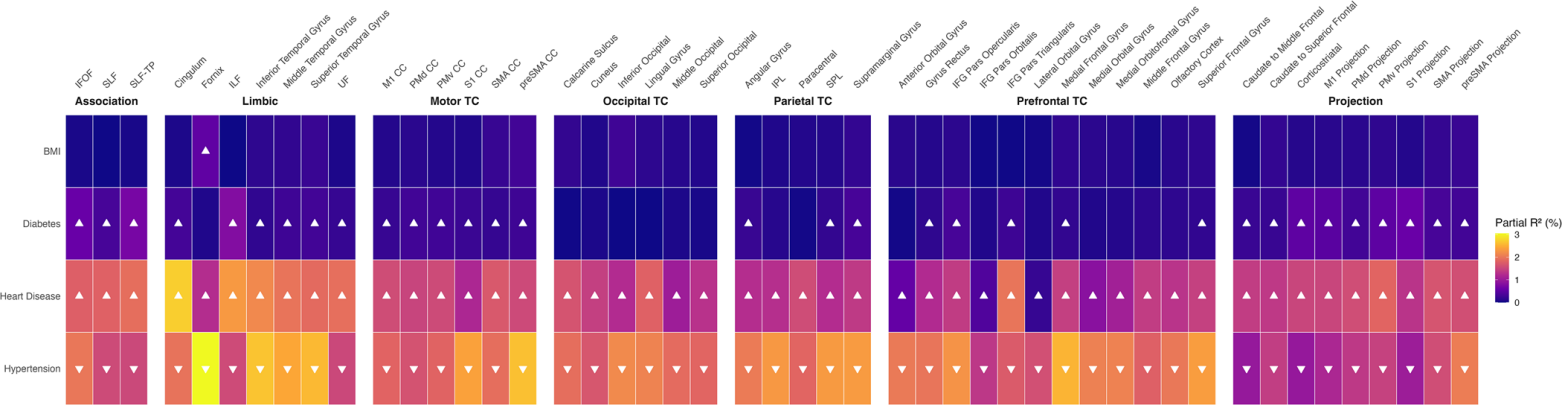
Associations between VRFs and FA_FWcorr_. Reduced models of the primary regression models were used to quantify the relative contributions of VRFs to alterations in FA_FWcorr_ across the 48 white matter tracts. The heatmap illustrates partial *R* ^2^ values, highlighting the relative contributions to FA_FWcorr_, from the 5‐year restricted primary analysis. Arrows indicate direction of association, with upward‐pointing arrows indicating a positive beta and downward‐pointing arrows indicating a negative beta. *Tracts surviving correction for multiple comparisons. CC, corpus callosum; FW, free water; FA_FWcorr_, FW‐corrected fractional anisotropy; IFG, inferior frontal gyrus; IFOF, inferior fronto‐occipital fasciculus; ILF, inferior longitudinal fasciculus; IPL, inferior parietal lobe; M1, primary motor cortex; PMd, dorsal premotor cortex; PMv, ventral premotor; S1, primary somatosensory cortex; SLF, superior longitudinal fasciculus; SLF‐TP, temporoparietal superior longitudinal fasciculus; SMA, supplementary motor area; SPL, superior parietal lobe; TC, transcallosal; UF, uncinate fasciculus.

VRFs exhibited different associations with FA_FWcorr_ within the white matter tracts (Figure 3). Hypertension showed widespread, significant associations with lower FA_FWcorr_ across 48 tracts. The most pronounced effects were in the fornix (partial *R* ^2^ = 2.9%, *p_FDR_ *< 0.001), pre‐supplementary motor area (SMA) TC (partial *R* ^2^ = 2.6%, *p_FDR_ *< 0.001), and inferior temporal gyrus TC tract (partial *R* ^2^ = 2.5%, *p_FDR_ *< 0.001). Similarly, heart disease showed widespread, significant associations with FA_FWcorr_ within 48 tracts, but heart disease was associated with higher FA_FWcorr_, with the strongest effects in the cingulum (partial *R* ^2^ = 2.6%, *p_FDR_ *< 0.001), ILF (partial *R* ^2^ = 2.0%, *p_FDR_ *< 0.001), and inferior temporal gyrus TC tract (partial *R* ^2^ = 1.9%, *p_FDR_ *< 0.001). In contrast, diabetes associated with 32 tracts, including with higher FA_FWcorr_, strongest in the ILF (partial *R^2^* = 0.73%, *p_FDR_ *< 0.001), temporoparietal superior longitudinal fasciculus (SLF‐TP) (partial *R* ^2^ = 0.65%, *p_FDR_ *< 0.001), and S1 projection (partial *R* ^2^ = 0.58%, *p_FDR_ *< 0.001). BMI showed a single significant association in the fornix (partial *R* ^2^ = 0.51%, *p_FDR_* = 0.005), with higher BMI associated with higher FA_FWcorr_. Refer to Figure S4 for a forest plot and Table S4 for the strongest statistical results.

In the 1‐year restricted analysis, the magnitude of effects for hypertension and heart disease remained consistent. In contrast, effect estimates for diabetes were attenuated and no longer reached statistical significance, whereas the effects of BMI appeared stronger, with 15 tracts reaching significance. See Figure S5 for a partial *R* ^2^ heatmap and Figure S6 for a forest plot from the 1‐year restricted analysis.

### 3.3 VRFs and MD_FWcorr_

**FIGURE 4:**
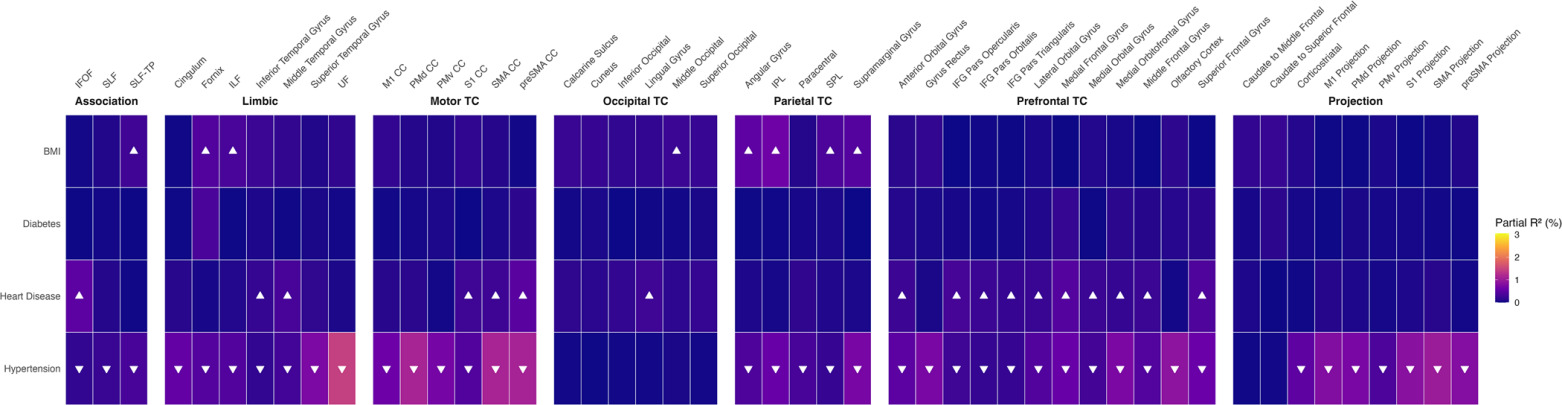
Associations between VRFs and MD_FWcorr_. Reduced models of the primary regression models were used to quantify the relative contributions of VRFs to alterations in MD_FWcorr_ across the 48 white matter tracts. The heatmap illustrates partial *R* ^2^ values, highlighting the relative contributions to MD_FWcorr_, from the 5‐year restricted primary analysis. Arrows indicate direction of association, with upward‐pointing arrows indicating a positive beta and downward‐pointing arrows indicating a negative beta. *Tracts surviving correction for multiple comparisons. CC, corpus callosum; IFG, inferior frontal gyrus; IFOF, inferior fronto‐occipital fasciculus; ILF, inferior longitudinal fasciculus; IPL, inferior parietal lobe; M1, primary motor cortex; MD_FWcorr_, FW‐corrected mean diffusivity; PMd, dorsal premotor cortex; PMv, ventral premotor; S1, primary somatosensory cortex; SLF, superior longitudinal fasciculus; SLF‐TP, temporoparietal superior longitudinal fasciculus; SMA, supplementary motor area; SPL, superior parietal lobe; TC, transcallosal; UF, uncinate fasciculus; VRF, vascular risk factor.

VRFs also demonstrated unique associations with MD_FWcorr_ within the white matter tracts (Figure 4). Hypertension demonstrated the most widespread effects, associated with MD_FWcorr_ in 40 tracts, with hypertension associated with lower MD_FWcorr_. The strongest associations for hypertension were in the uncinate fasciculus (partial *R* ^2^ = 1.4%, *p_FDR_ *< 0.001), dorsal premotor cortex TC (partial *R* ^2^ = 1.2%, *p_FDR_ *< 0.001), and SMA TC (partial *R* ^2^ = 1.2%, *p_FDR_ *< 0.001). In contrast, heart disease, BMI, and diabetes exhibited more limited associations. Heart disease associated with MD_FWcorr_ in 17 tracts, with heart disease associated with higher MD_FWcorr_, strongest in the medial frontal gyrus (partial *R* ^2^ = 0.46%, *p_FDR_* = 0.0062), pre‐SMA TC tract (partial *R* ^2^ = 0.45%, *p_FDR_* = 0.0062), and superior frontal gyrus TC (partial *R* ^2^ = 0.43%, *p_FDR_* = 0.0062). BMI associated with MD_FWcorr_ in eight tracts, with higher BMI associated with higher MD_FWcorr_, strongest in the IPL TC tract (partial *R* ^2^ = 0.64%, *p_FDR_ *< 0.001), angular gyrus TC (partial *R* ^2^ = 0.51%, *p_FDR_* = 0.0026), and supramarginal gyrus TC (partial *R* ^2^ = 0.41%, *p_FDR_* = 0.0079). Diabetes associated with lower MD_FWcorr_ in one tract, the fornix (partial *R* ^2^ = 0.35%, *p_FDR_* = 0.063), but did not survive FDR correction. See Figure S7 for a forest plot and Table S5 for the strongest statistical results.

In the 1‐year restricted analysis, no hypertension or diabetes association reached significance, whereas only one association for BMI remained significant. Effect estimates for hypertension and diabetes were attenuated and did not reach statistical significance. In contrast, the magnitude of associations for heart disease remained consistent, with 10 tracts maintaining statistical significance. See Figure S8 for a partial *R* ^2^ heatmap and Figure S9 for a forest plot from the 1‐year restricted analysis.

### 3.4 VRFs and AxD_FWcorr_

**FIGURE 5:**
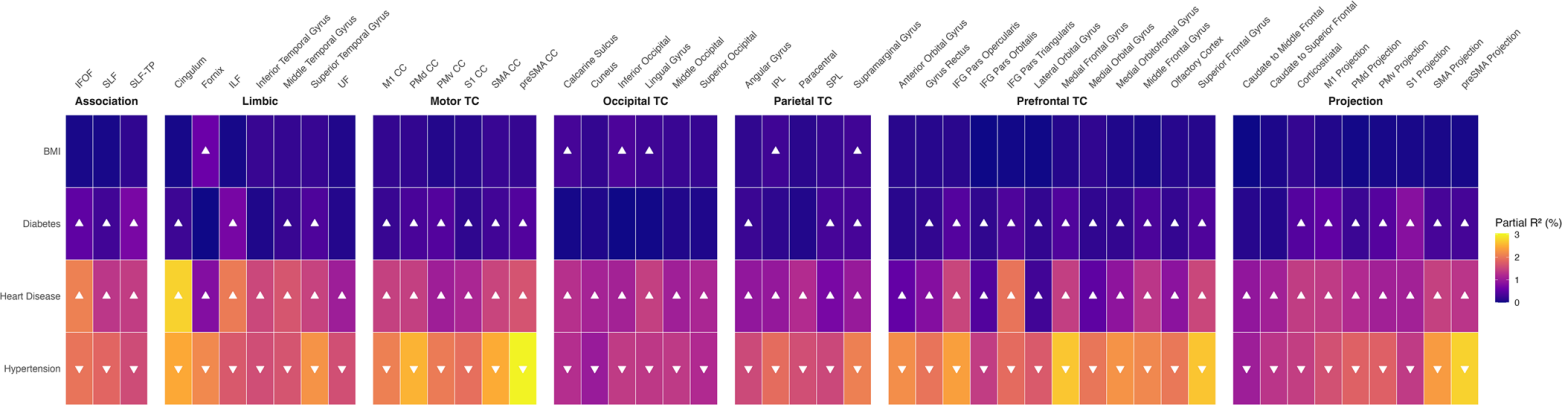
Associations between VRFs and AxD_FWcorr_. Reduced models of the primary regression models were used to quantify the relative contributions of VRFs to alterations in AxD_FWcorr_ across the 48 white matter tracts. The heatmap illustrates partial *R* ^2^ values, highlighting the relative contributions to AxD_FWcorr_, from the 5‐year restricted primary analysis. Arrows indicate direction of association, with upward‐pointing arrows indicating a positive beta and downward‐pointing arrows indicating a negative beta. *Tracts surviving correction for multiple comparisons. AxD_FWcorr_, FW‐corrected axial diffusivity; CC, corpus callosum; IFG, inferior frontal gyrus; IFOF, inferior fronto‐occipital fasciculus; ILF, inferior longitudinal fasciculus; IPL, inferior parietal lobe; M1, primary motor cortex; PMd, dorsal premotor cortex; PMv, ventral premotor; S1, primary somatosensory cortex; SLF, superior longitudinal fasciculus; SLF‐TP, temporoparietal superior longitudinal fasciculus; SMA, supplementary motor area; SPL, superior parietal lobe; TC, transcallosal; UF, uncinate fasciculus; VRF, vascular risk factor.

Like FA_FWcorr_, hypertension showed widespread associations with lower AxD_FWcorr_ across 48 tracts (Figure 5). The strongest associations were in the pre‐SMA TC tract (partial *R* ^2^ = 2.9%, *p_FDR_ *< 0.001), pre‐SMA projection (partial *R* ^2^ = 2.6%, *p_FDR_ *< 0.001), and medial frontal gyrus TC (partial *R* ^2^ = 2.6%, *p_FDR_ *< 0.001). Similarly, heart disease showed widespread, significant associations with FA_FWcorr_ within 48 tracts, but heart disease was associated with higher AxD_FWcorr_, with the most pronounced associations in the cingulum (partial *R* ^2^ = 2.6%, *p_FDR_ *< 0.001), ILF (partial *R* ^2^ = 1.8%, *p_FDR_ *< 0.001), and inferior frontal gyrus pars triangularis TC (partial *R* ^2^ = 1.7%, *p_FDR_ *< 0.001). In contrast, diabetes exhibited associations with 34 tracts and with lower AxD_FWcorr_, strongest in the S1 projection (partial *R* ^2^ = 0.82%, *p_FDR_ *< 0.001), SLF‐TP (partial *R* ^2^ = 0.67%, *p_FDR_ *< 0.001), and ILF (partial *R* ^2^ = 0.65%, *p_FDR_ *< 0.001). Higher BMI showed six significant associations with higher AxD_FWcorr_, with the strongest in the fornix (partial *R* ^2^ = 0.62%, *p_FDR_ *< 0.001), inferior occipital TC (partial *R* ^2^ = 0.36%, *p_FDR_* = 0.027), and calcarine sulcus (partial *R* ^2^ = 0.31%, *p_FDR_* = 0.037). See Figure S10 for a forest plot and Table S6 for the strongest statistical results.

In the 1‐year restricted analysis, the associations for hypertension and heart disease remained significant, with their magnitude of effects remaining consistent. In contrast, all 28 associations for diabetes were no longer significant, and effect estimates were attenuated. Associations for BMI emerged, increasing from one to 14 tracts, with the effects of BMI appearing stronger. See Figure S11 for a partial *R* ^2^ heatmap and Figure S12 for a forest plot from the 1‐year restricted analysis.

### 3.5 VRFs and RD_FWcorr_

**FIGURE 6:**
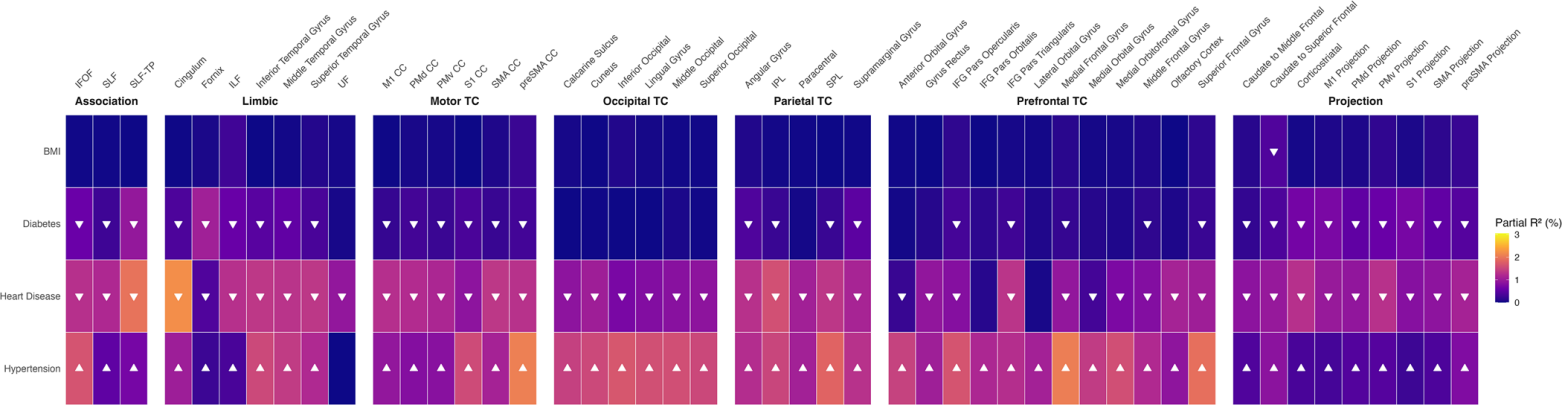
Associations between VRFs and RD_FWcorr_. Reduced models of the primary regression models were used to quantify the relative contributions of VRFs to alterations in RD_FWcorr_ across the 48 white matter tracts. The heatmap illustrates partial R^2^ values, highlighting the relative contributions to RD_FWcorr_, from the 5‐year restricted primary analysis. Arrows indicate direction of association, with upward‐pointing arrows indicating a positive beta and downward‐pointing arrows indicating a negative beta. *Tracts surviving correction for multiple comparisons. CC, corpus callosum; IFG, inferior frontal gyrus; IFOF, inferior fronto‐occipital fasciculus; ILF, inferior longitudinal fasciculus; IPL, inferior parietal lobe; M1, primary motor cortex; PMd, dorsal premotor cortex; PMv, ventral premotor; RD_FWcorr_, radial diffusivity; S1, primary somatosensory cortex; SLF, superior longitudinal fasciculus; SLF‐TP, temporoparietal superior longitudinal fasciculus; SMA, supplementary motor area; SPL, superior parietal lobe; TC, transcallosal; UF, uncinate fasciculus; VRF, vascular risk factor.

Associations between VRFs and RD_FWcorr_ were observed within the 48 white matter tracts (Figure 6). Hypertension and heart disease showed the most widespread, significant associations with RD_FWcorr_, whereas diabetes demonstrated more limited associations, and BMI only associated with one tract. Hypertension associated with 47 tracts, with hypertension associated with higher RD_FWcorr_, most strongly in the pre‐SMA TC tract (partial *R* ^2^ = 2.0%, *p_FDR_ *< 0.001), medial frontal gyrus TC (partial *R* ^2^ = 2.0%, *p_FDR_ *< 0.001), and superior frontal gyrus TC (partial *R* ^2^ = 1.8%, *p_FDR_ *< 0.001). Heart disease associated with 46 tracts, but it was associated with lower RD_FWcorr_ and most strongly associated with RD_FWcorr_ in the cingulum (partial *R* ^2^ = 2.1%, *p_FDR_ *< 0.001), SLF‐TP (partial *R* ^2^ = 1.8%, *p_FDR_ *< 0.001), and IPL TC tract (partial *R* ^2^ = 1.4%, *p_FDR_ *< 0.001). Diabetes associated with 32 tracts and with higher RD_FWcorr_, with the strongest associations in the fornix (partial *R* ^2^ = 1.1%, *p_FDR_ *< 0.001), SLF‐TP (partial *R* ^2^ = 0.91%, *p_FDR_ *< 0.001), and M1 projection (partial *R* ^2^ = 0.72%, *p_FDR_ *< 0.001). Higher BMI showed a single significant association with higher RD_FWcorr_ in the caudate to superior frontal gyrus (partial *R* ^2^ = 0.40%, *p_FDR_* = 0.029). See Figure S13 for a forest plot and Table S7 for the strongest statistical results.

In the 1‐year restricted analysis, only associations for hypertension in 47 tracts and associations for heart disease in 48 tracts remained significant, with comparable effect sizes. No associations reached significance for diabetes or BMI. See Figure S14 for a partial *R* ^2^ heatmap and Figure S15 for a forest plot from the 1‐year restricted analysis.

## 4 DISCUSSION

In this large, multi‐cohort study, we examined the differential contributions of four major VRFs to white matter microstructure across 48 tracts. In the study, hypertension and heart disease demonstrated the most robust and widespread independent associations, particularly in limbic and commissural tracts. Specifically, hypertension was associated with a pattern of microstructure in the fornix and pre‐SMA TC tract, while heart disease exhibited strong associations with the cingulum and ILF. In contrast, diabetes and BMI displayed weaker and fewer effects on white matter tracts. While diabetes was associated with microstructural alterations in projection and association tracts, these associations were less robust than the widespread effects observed with hypertension and heart disease, as they did not persist in stricter sensitivity analyses. Collectively, these results highlight that hypertension and heart disease are the strongest VRF correlates of white matter microstructure.

The most widespread associations were demonstrated by hypertension, which persisted across both the 5‐ and 1‐year restricted analyses. Across white matter tracts, hypertension associated with increased FW, suggesting increased extracellular fluid (possibly due to neurodegeneration or inflammation^35^); decreased FA_FWcorr_, suggesting decreased fiber coherence^36^; decreased AxD_FWcorr_, suggesting increased axonal degeneration^37^, ^38^; and increased RD_FWcorr_, suggesting greater demyelination.^39^, ^40^ These findings are consistent with prior work demonstrating a link between hypertension and reduced white matter microstructure.^41^, ^42^ Notably, these effects were strongest in the fornix and pre‐SMA TC tract, suggesting that hypertension may disproportionately target tracts involved in memory and higher motor control.^43^, ^44^, ^45^, ^46^ The fornix and anterior callosal regions are perfused by small perforating branches of the anterior cerebral artery and anterior communicating artery.^47^ Perforating arteries are particularly susceptible to hypertensive injury because they have limited collateral circulation and are prone to arteriolosclerosis.^48^ The pervasiveness of these associations, which persisted even in our strictly temporally matched sensitivity analysis, highlights the strong, independent association between hypertension and global microstructure.

Heart disease also exhibited unique associations with white matter microstructure; however, these associations were paradoxical, associating with increased FA_FWcorr_, increased AxD_FWcorr_, and decreased RD_FWcorr_ across white matter tracts, with varying or not statistically significant effects on MD_FWcor_ in select white matter tracts and decreased FW in one white matter tract. While the directions of these associations typically indicate improved white matter integrity, in the context of vascular pathology, they more likely represent complex alterations in microstructure.^49^, ^50^ Other DTI studies also demonstrated counterintuitive findings in other neurodegenerative contexts, including AD,^51^, ^52^ multiple sclerosis,^53^ Huntington's disease,^54^ and traumatic brain injury,^55^, ^56^ which are typically attributed to mechanisms such as gliosis (which can restrict water diffusion via cellular proliferation or hypertrophy)^51^ or selective degeneration of crossing fibers (which can alter tract geometry).^54^, ^57^ Notably, heart disease exhibited the strongest effect sizes in the cingulum and ILF. While the mechanisms linking heart disease to brain health are multifactorial, heart disease frequently leads to reduced cardiac output and chronic systemic hypoperfusion, which can lead to reductions in cerebral blood flow.^58^ Reduced cerebral perfusion disproportionately affects distal vascular “watershed” territories, where perfusion pressure is lowest.^59^ Internal watershed regions are located within the deep periventricular and centrum semiovale white matter, through which long association fibers such as the cingulum^60^ and ILF^61^ run, rendering these tracts particularly vulnerable to hypoperfusion and small‐vessel disease. Despite the counterintuitive directionality, these signals were highly robust across our sensitivity analyses. This paradoxical relationship between heart disease and white matter microstructure highlights the need for replication in independent cohorts and warrants further investigation into the underlying mechanisms.

In contrast to hypertension and heart disease, diabetes exhibited less robust associations with white matter microstructure, as these effects did not persist in the 1‐year analysis. Diabetes was primarily associated with projection and association tracts and, specifically, the ILF and SLF‐TP. While diabetes related to increased RD_FWcorr_ in numerous tracts, these associations were not robust. The attenuation of effect estimates and loss of statistical significance in our stricter sensitivity analysis suggests that diabetes associations with white matter microstructure may be less robust compared to hypertension or that its association is mediated through shared vascular pathways or comorbidities not fully captured in these models.

Our analysis revealed that BMI most strongly linked to microstructure in the fornix. BMI associations were also consistent with the “obesity paradox” observed in older adults,^62^, ^63^ where higher BMI related to DTI metrics was typically interpreted as indicative of stronger white matter integrity (e.g., increased FA_FWcorr_ and AxD_FWcorr_). While this relationship is counterintuitive for VRFs, it is possible that a higher BMI has a protective effect in older adults. Several studies also report that a higher BMI has a positive effect on brain health and cognition,^64^, ^65^, ^66^, ^67^ often interpreted within the context of an “obesity paradox” possibly driven by differences in nutritional or metabolic reserve.^68^ Conversely, other studies find that a higher BMI is linked to poorer brain outcomes^69^, ^70^, ^71^ or follow a U‐shaped relationship,^72^, ^73^ which may explain the positive associations observed here; however, future studies are needed to clarify these associations.

This study includes several strengths. First, we used a large, multi‐site, harmonized dataset. Second, we implemented a comprehensive methodology with 48 tract templates and advanced FW‐corrected DTI metrics. Third, we used FW‐corrected DTI metrics, enabling more accurate assessment of white matter microstructure by reducing the impact of partial volume effects. A fourth strength is our 1‐year sensitivity analysis, which allowed us to evaluate the consistency of our effect estimates within a stricter temporal window, minimizing potential bias from the interval between assessments. Conversely, this study has several limitations. First, VRF variables represent lifetime history or current diagnosis, not severity, duration, or management status. For example, participants with “hypertension” could range from those with well‐managed blood pressure on medication to those with uncontrolled hypertension. This heterogeneity is a source of unmeasured variance. The detection of robust associations, particularly for hypertension and heart disease, despite this variability emphasizes the powerful impact of these conditions; however, future studies using more precise physiological measures (e.g., systolic blood pressure or HbA_1c_ levels) are needed to parse these more granular effects. Second, although VRF assessments closest to the DTI scan were used in the analysis, including cases years apart could potentially introduce exposure misclassification and preclude causal inferences. For example, a participant can develop a VRF after the clinical assessment but before MRI scan, leading to their misclassification as “unexposed” and a likely underestimation of the true association. Conversely, a participant may develop a VRF after the DTI scan, making it difficult to determine the temporal sequence of events (i.e., whether the white matter changes preceded or followed the onset of the risk factor). Third, while some cohorts utilized laboratory data and medication inspections to verify diagnoses, others relied on self‐report, which might have introduced misclassification bias or unmeasured variance in the accuracy of VRF status across the pooled sample. Fourth, the management of VRFs, such as medication adherence, was not accounted for and might have confounded the VRF effects on white matter microstructure. Fifth, participants able to complete a MRI scan may represent a somewhat healthier subset of the cohort, which could introduce survivor bias and potentially lead to an underestimation of the true impact of these VRFs, contributing to the observed paradoxical associations. Sixth, while our analysis controlled for cognitive status, neurodegenerative diseases beyond AD were not included in this study. As such, these findings are specific to the AD continuum and may not generalize to individuals with neurodegenerative conditions characterized by other pathophysiological pathways. Finally, demographic heterogeneity across cohorts may contribute to the observed differences in white matter microstructure, limiting the generalizability of the findings.

In conclusion, our findings highlight the robust and independent associations of hypertension and heart disease with white matter tract microstructure, whereas BMI and diabetes demonstrated weaker independent effects. The widespread associations that hypertension and heart disease exhibit with white matter microstructure emphasize their deleterious effects on white matter tract integrity, while the paradoxical associations with heart disease warrant further investigation. Among VRFs, hypertension and heart disease explain the greatest unique variance in white matter microstructure. Although these risk factors manifest through divergent microstructural patterns, their robust and widespread effects suggest they may be particularly influential in cerebrovascular pathways affecting brain health and cognition. This study indicates that prioritizing interventions for hypertension and heart disease may more reliably yield greater benefits in preserving white matter integrity compared to BMI and diabetes.

## CONFLICT OF INTEREST STATEMENT

T.J.H. is on the scientific advisory board for Vivid Genomics, serves as a consultant for Circular Genomics, serves as deputy editor for *Alzheimer's & Dementia: TRCI*, and serves as senior associate editor for *Alzheimer's & Dementia*. A.L.J. serves as Chair of the Observational Study Monitoring Board for the Diverse‐VCID study and has served as an advisor for Lantheus – Diagnostic and Therapeutic Innovations. S.C.J. has received consulting fees from Merck, Eli Lilly, and Enigma Biomedical and serves on scientific advisory boards for Alamar, AlzPath, Sunbird Bio, Neurocode Labs, and Lantheus. B.A.L. serves as editor in chief *of SPIE Journal of Medical Imaging*. L.L.B. serves as deputy editor for *Alzheimer's & Dementia*. W.K. serves on external advisory boards for the University of Kansas, Boston University, and Mt. Sinai Alzheimer's Disease Research Centers. K.G.S. has received consulting fees from Mindset Integrated Co. and the University of California, Berkeley. J.M. has received honoraria from the Concussion Legacy Foundation, Imperial College London, and the Lou Ruvo Center for Brain Health. J.D.L., Y.V., A.S., N.S., K.R.P., Y.Y., A.D., P.K., M.E.K., C.G., N.R.N., K.R., N.M.K., Z.L., T.Y., S.L.R., P.Z., A.J.L., A.M.B., R.M., S.A.B., B.B.B., J.S., D.A.B., A.J.S., M.L.C., D.B.A., ADNI, ADSP, and ADSP‐PHC have no conflicts to disclose. Author disclosures are available in the Supporting Information.

## CONSENT STATEMENT

All participants provided informed consent in their respective cohort studies.

## Supporting information



Supporting Information

Supporting Information
