# Supplementary material for: The role of vascular risk factors in white matter tract microstructure: a multi‐cohort study in older adults
Source: Alzheimers Dement. 2026 Jul 31;22(8):e71698. doi: 10.1002/alz.71698 (PMC13425621; doi:10.1002/alz.71698)
Supplement: Supplementary file 1 — Supporting Information [file ALZ-22-e71698-s001.docx]

| Supplementary Table 2. Strongest VRF Associations with FW | | | | | |
| --- | --- | --- | --- | --- | --- |
| Tract | **Tract Type** | **Partial R^2^** | **β (95% CI)** | **p-value** | **FDR-corrected p-value** |
| *BMI* |  |  |  |  |  |
| Cingulum | Limbic | 0.52% | –0.00042 (–0.00063, –0.00021) | 9.48×10^-5^ | 0.0046* |
| ILF | Limbic | 0.41% | –0.00045 (–0.00070, –0.00019) | 0.00053 | 0.013* |
| Fornix | Limbic | 0.37% | –0.00048 (–0.00077, –0.00020) | 0.00098 | 0.013* |
| *Diabetes* |  |  |  |  |  |
| Cingulum | Limbic | 0.21% | –0. 0034 (–0.0061, –0.0008) | 0.012 | 0.20 |
| UF | Limbic | 0.21% | –0.0048 (–0.0086, –0.0010) | 0.014 | 0.20 |
| IFG Pars Opercularis | Prefrontal TC | 0.20% | –0.0048 (–0.0086, –0.0009) | 0.016 | 0.20 |
| *Heart Disease* |  |  |  |  |  |
| Fornix | Limbic | 0.28% | –0.0053 (–0.0090, –0.0017) | 0.0044 | 0.13 |
| Cingulum | Limbic | 0.26% | –0.0038 (–0.0064, –0.0011) | 0.0053 | 0.13 |
| UF | Limbic | 0.17% | –0.0042 (–0.0081, –0.0005) | 0.027 | 0.35 |
| *Hypertension* |  |  |  |  |  |
| IPL | Parietal TC | 2.5% | 0.017 (0.013, 0.021) | 7.45×10^-18^ | 3.57×10^-16^* |
| Angular Gyrus | Parietal TC | 2.4% | 0.017 (0.013, 0.021) | 1.85×10^-17^ | 4.43×10^-16^* |
| Supramarginal Gyrus | Parietal TC | 2.3% | 0.015 (0.011, 0.019) | 2.42×10^-16^ | 3.87×10^-15^* |
| Note. Statistical outputs are provided for the white matter tracts in which each VRF exhibited the greatest partial R^2^. These tracts represent where each VRF uniquely explained the most variance compared to other tracts from the 5-year restricted primary analysis. *FDR-corrected p-value < 0.05. IFG, inferior frontal gyrus; ILF, inferior longitudinal fasciculus; IPL, inferior parietal lobule; TC, transcallosal; UF, uncinate fasciculus. | | | | | |

| **Supplementary Table 3**. Participant Characteristics from the 1-Year Restricted Analysis | | | | | | |
| --- | --- | --- | --- | --- | --- | --- |
| **Measure** | **Cohort** | | | | | |
|  | **ADNI** | **NACC** | **ROS/MAP/MARS** | **VMAP** | **WRAP** | **Total** |
| *Cohort Characteristics* | | | | | | |
| Total number of participants | 668 | 143 | 134 | 526 | 17 | 1488 |
| *Demographic Characteristics* | | | | | | |
| Age (years) | 74.19±8.12 | 75.60±9.37 | 84.36±7.95 | 70.86±9.13 | 64.36±6.44 | 73.95±9.38 |
| Absolute interval between DTI and VRF assessment (years) | 0.11±0.28 | 0.24±0.41 | 0.45±0.48 | 0.34±0.26 | 0±0 | 0.23±0.33 |
| Sex (% female) | 50.90 | 57.34 | 72.39 | 49.62 | 64.71 | 53.16 |
| Education (years) | 16.31±2.42 | 13.82±4.31 | 15.78±3.13 | 16.08±2.49 | 16.65±2.91 | 15.94±2.84 |
| Race (% non-Hispanic White) | 75.60 | 65.73 | 80.60 | 85.55 | 100 | 78.90 |
| Cognitive status at baseline (% cognitively unimpaired) | 42.66 | 46.85 | 70.90 | 76.81 | 94.12 | 58.27 |
| Systolic blood pressure (mmHg) | 133.06±16.95 | 138.55±19.78 | 128.88±19.78 | 135.25±17.42 | 126.71±18.90 | 133.90±17.83 |
| *Vascular Risk Factors* | | | | | | |
| Hypertension (%) | 44.91 | 83.92 | 92.54 | 63.45 | 41.18 | 59.81 |
| Diabetes (%) | 63.77 | 30.77 | 23.88 | 15.78 | 5.88 | 39.38 |
| Heart disease (%) | 62.28 | 20.28 | 26.12 | 5.51 | 23.53 | 34.48 |
| Body mass index (kg/m^2^) | 27.43±5.24 | 27.92±5.57 | 26.56±4.94 | 27.68±4.87 | 28.09±6.16 | 27.49±5.13 |
| **Note.** Data reflect the sample included in the 1-year restricted secondary analysis. Values denoted as mean±standard deviation or frequency. Abbreviations: ADNI, Alzheimer's Disease Neuroimaging Initiative; NACC, National Alzheimer's Coordinating Center; ROS, Religious Orders Study; MAP, Rush Memory and Aging Project; MARS, Minority Aging Research Study; VMAP, Vanderbilt Memory & Aging Project; WRAP, Wisconsin Registry for Alzheimer's Prevention. | | | | | | |

| Supplementary Table 4. Strongest VRF Associations with FA_FWcorr_ | | | | | |
| --- | --- | --- | --- | --- | --- |
| Tract | **Tract Type** | **Partial R^2^** | **β (95% CI)** | **p-value** | **FDR-corrected p-value** |
| *BMI* |  |  |  |  |  |
| Fornix | Limbic | 0.51% | 0.00047 (0.00023, 0.00070) | 0.00011 | 0.0051* |
| preSMA TC | Motor TC | 0.29% | 0.00045 (0.00015, 0.00076) | 0.0033 | 0.079 |
| Inferior Occipital | Occipital TC | 0.26% | 0.00040 (0.00012, 0.00069) | 0.0057 | 0.09 |
| *Diabetes* |  |  |  |  |  |
| ILF | Limbic | 0.73% | 0.0065 (0.0037, 0.0092) | 3.58×10^-6^ | 0.00017* |
| SLF-TP | Association | 0.65% | 0.0061 (0.0033, 0.0088) | 1.22×10^-5^ | 0.00029* |
| S1 Projection | Projection | 0.58% | 0.0064 (0.0034, 0.0095) | 3.69×10^-5^ | 0.00059* |
| *Heart Disease* |  |  |  |  |  |
| Cingulum | Limbic | 2.6% | 0.013 (0.010, 0.016) | 4.02×10^-23^ | 2.06×10^-21^* |
| ILF | Limbic | 2.0% | 0.011 (0.008, 0.014) | 2.09×10^-19^ | 5.02×10^-18^* |
| Inferior Temporal Gyrus | Limbic | 1.9% | 0.012 (0.0086, 0.015) | 1.02×10^-13^ | 1.64×10^-12^* |
| *Hypertension* |  |  |  |  |  |
| Fornix | Limbic | 2.9% | –0.014 (–0.016, –0.011) | 9.91×10^-21^ | 4.76×10^-19^* |
| preSMA TC | Motor TC | 2.6% | –0.016 (–0.020, –0.013) | 2.30×10^-18^ | 5.52×10^-17^* |
| Inferior Temporal Gyrus | Limbic | 2.5% | –0.012 (–0.016, –0.010) | 4.83×10^-18^ | 7.73×10^-17^* |
| Note. Statistical outputs are provided for the white matter tracts in which each VRF exhibited the greatest partial R^2^. These tracts represent where each VRF uniquely explained the most variance in FA_FWcorr_ compared to other tracts from the 5-year restricted primary analysis. *FDR-corrected p-value < 0.05. ILF, inferior longitudinal fasciculus; S1, primary somatosensory cortex; SLF-TP, temporoparietal superior longitudinal fasciculus; SMA, supplementary motor area; TC, transcallosal. | | | | | |

| Supplementary Table 5. Strongest VRF Associations with MD_FWcorr_ | | | | | |
| --- | --- | --- | --- | --- | --- |
| Tract | **Tract Type** | **Partial R^2^** | **β (95% CI)** | **p-value** | **FDR-corrected p-value** |
| *BMI* |  |  |  |  |  |
| IPL | Parietal TC | 0.64% | 2.07×10^-7^ (1.14×10^-7^, 3.00×10^-7^) | 1.34×10^-5^ | 0.00064* |
| Angular Gyrus | Parietal TC | 0.51% | 1.86×10^-7^ (9.19×10^-8^, 2.80×10^-7^) | 0.00011 | 0.0026* |
| Supramarginal Gyrus | Parietal TC | 0.41% | 1.36×10^-7^ (5.92×10^-8^, 2.13×10^-7^) | 0.00053 | 0.0079* |
| *Diabetes* |  |  |  |  |  |
| Fornix | Limbic | 0.35% | –3.77×10^-6^ (–6.07×10^-6^, –1.47×10^-6^) | 0.0013 | 0.063 |
| Medial Frontal Gyrus | Prefrontal TC | 0.15% | 1.04×10^-6^ (7.89×10^-8^, 2.00×10^-6^) | 0.034 | 0.54 |
| Caudate to Superior Frontal | Prefrontal TC | 0.13% | –7.48×10^-7^ (–1.51×10^-6^, 1.07×10^-8^) | 0.053 | 0.54 |
| *Heart Disease* |  |  |  |  |  |
| Medial Frontal Gyrus | Prefrontal TC | 0.46% | 1.80×10^-6^ (8.46×10^-7^, 2.75×10^-6^) | 0.00022 | 0.0062* |
| preSMA TC | Motor TC | 0.45% | 1.92×10^-6^ (8.89×10^-7^, 2.94×10^-6^) | 0.00026 | 0.0062* |
| Superior Frontal Gyrus | Parietal TC | 0.43% | 1.45×10^-6^ (6.51×10^-7^, 2.25×10^-6^) | 0.00039 | 0.0062* |
| *Hypertension* |  |  |  |  |  |
| UF | Limbic | 1.4% | –3.30×10^-6^ (–4.31×10^-6^, –2.29×10^-6^) | 1.42×10^-10^ | 6.84×10^-9^* |
| PMd TC | Motor TC | 1.2% | –3.12×10^-6^ (–4.14×10^-6^, –2.10×10^-6^) | 1.95×10^-9^ | 4.67×10^-8^* |
| SMA TC | Motor TC | 1.2% | –3.25×10^-6^ (–4.33×10^-6^, –2.17×10^-8^) | 4.43×10^-9^ | 7.08×10^-8^* |
| Note. Statistical outputs are provided for the white matter tracts in which each VRF exhibited the greatest partial R^2^. These tracts represent where each VRF uniquely explained the most variance in MD_FWcorr_ compared to other tracts from the 5-year restricted primary analysis. *FDR-corrected p-value < 0.05. IPL, inferior parietal lobule; PMd, dorsal premotor; SMA, supplementary motor area; TC, transcallosal; UF, uncinate fasciculus. | | | | | |

| Supplementary Table 6. Strongest VRF Associations with AxD_FWcorr_ | | | | | |
| --- | --- | --- | --- | --- | --- |
| Tract | **Tract Type** | **Partial R^2^** | **β (95% CI)** | **p-value** | **FDR-corrected p-value** |
| *BMI* |  |  |  |  |  |
| Fornix | Limbic | 0.62% | 7.60×10^-7^ (4.12×10^-7^, 1.11×10^-6^) | 1.92×10^-5^ | 9.20×10^-5^* |
| Inferior Occipital | Occipital TC | 0.36% | 7.62×10^-7^ (304×10^-7^, 1.22×10^-6^) | 0.0011 | 0.027* |
| Calcarine Sulcus | Occipital TC | 0.31% | 6.37×10^-7^ (2.28×10^-7^, 1.05×10^-6^) | 0.0023 | 0.037* |
| *Diabetes* |  |  |  |  |  |
| S1 Projection | Projection | 0.82% | 9.51×10^-6^ (5.75×10^-6^, 1.33×10^-5^) | 7.85×10^-7^ | 3.77×10^-5^* |
| SLF-TP | Association | 0.67% | 6.89×10^-6^ (3.85×10^-6^, 9.93×10^-6^) | 8.96×10^-6^ | 0.00018* |
| ILF | Limbic | 0.65% | 6.78×10^-6^ (3.76×10^-6^, 9.80×10^-6^) | 1.13×10^-5^ | 0.00018* |
| *Heart Disease* |  |  |  |  |  |
| Cingulum | Limbic | 2.6% | 1.55×10^-5^ (1.20×10^-5^, 1.89×10^-5^) | 2.36×10^-18^ | 1.13×10^-16^* |
| ILF | Limbic | 1.8% | 1.13×10^-5^ (8.26×10^-6^, 1.43×10^-5^) | 2.25×10^-13^ | 5.41×10^-12^* |
| IFG Pars Triangularis | Prefrontal TC | 1.7% | 1.19×10^-5^ (8.66×10^-5^, 1.51×10^-5^) | 6.18×10^-13^ | 9.89×10^-12^* |
| *Hypertension* |  |  |  |  |  |
| preSMA TC | Motor TC | 2.9% | –2.34×10^-5^ (–2.83×10^-5^, –1.85×10^-5^) | 1.06×10^-20^ | 5.09×10^-19^* |
| preSMA Projection | Projection | 2.6% | –1.60×10^-5^ (–1.95×10^-5^, –1.24×10^-5^) | 9.94×10^-19^ | 2.39×10^-17^* |
| Medial Frontal Gyrus | Prefrontal TC | 2.6% | –1.88×10^-5^ (–2.30×10^-5^, –1.47×10^-5^) | 1.54×10^-18^ | 2.46×10^-17^* |
| Note. Statistical outputs are provided for the white matter tracts in which each VRF exhibited the greatest partial R^2^. These tracts represent where each VRF uniquely explained the most variance in AxD_FWcorr_ compared to other tracts from the 5-year restricted primary analysis. *FDR-corrected p-value < 0.05. IFG, inferior frontal gyrus; ILF, inferior longitudinal fasciculus; S1, primary somatosensory cortex; SLF-TP, temporoparietal superior longitudinal fasciculus; SMA, supplementary motor area; TC, transcallosal. | | | | | |

| Supplementary Table 7. Strongest VRF Associations with RD_FWcorr_ | | | | | |
| --- | --- | --- | --- | --- | --- |
| Tract | **Tract Type** | **Partial R^2^** | **β (95% CI)** | **p-value** | **FDR-corrected p-value** |
| *BMI* |  |  |  |  |  |
| Caudate to Superior Frontal | Prefrontal TC | 0.40% | –1.74×10^-7^ (–2.73×10^-7^, –7.46×10^-8^) | 0.00060 | 0.029* |
| ILF | Limbic | 0.27% | 1.65×10^-7^ (5.14×10^-8^, 2.79×10^-7^) | 0.0045 | 0.11 |
| preSMA TC | Motor TC | 0.25% | –2.16×10^-7^ (–3.72×10^-7^, –5.88×10^-8^) | 0.0071 | 0.11 |
| *Diabetes* |  |  |  |  |  |
| Fornix | Limbic | 1.1% | –6.12×10^-6^ (–8.24×10^-6^, –4.01×10^-6^) | 1.58×10^-8^ | 7.56×10^-7^* |
| SLF-TP | Association | 0.91% | –5.06×10^-6^ (–4.98×10^-6^, –2.26×10^-6^) | 2.01×10^-7^ | 4.82×10^-6^* |
| M1 Projection | Projection | 0.72% | –3.79×10^-6^ (–5.40×10^-6^, –2.18×10^-6^) | 4.02×10^-6^ | 6.43×10^-5^* |
| *Heart Disease* |  |  |  |  |  |
| Cingulum | Limbic | 2.1% | –6.52×10^-6^ (–8.12×10^-6^, –4.92×10^-6^) | 1.89×10^-15^ | 9.08×10^-14^* |
| SLF-TP | Association | 1.8% | –5.06×10^-6^ (–6.41×10^-6^, –3.71×10^-6^) | 2,60×10^-13^ | 6.25×10^-12^* |
| IPL | Parietal TC | 1.4% | –4.93×10^-6^ (–6.45×10^-6^, –3.42×10^-6^) | 1.98×10^-10^ | 3.17×10^-9^* |
| *Hypertension* |  |  |  |  |  |
| preSMA TC | Motor TC | 2.0% | 7.50×10^-6^ (5.62×10^-6^, 9.38×10^-6^) | 7.96×10^-15^ | 3.82×10^-13^* |
| Medial Frontal Gyrus | Prefrontal TC | 2.0% | 6.54×10^-6^ (4.88×10^-6^, 8.21×10^-6^) | 1.78×10^-14^ | 4.28×10^-13^* |
| Superior Frontal Gyrus | Parietal TC | 1.8% | 5.61×10^-6^ (4.12×10^-6^, 7.09×10^-6^) | 1.87×10^-13^ | 3.00×10^-12^* |
| Note. Statistical outputs are provided for the white matter tracts in which each VRF exhibited the greatest partial R^2^. These tracts represent where each VRF uniquely explained the most variance in RD_FWcorr_ compared to other tracts from the 5-year restricted primary analysis. *FDR-corrected p-value < 0.05. ILF, inferior longitudinal fasciculus; IPL, inferior parietal lobe; M1, primary motor cortex; SLF-TP, temporoparietal superior longitudinal fasciculus; SMA, supplementary motor area; TC, transcallosal. | | | | | |

**Supplementary Figure 1. Beta Coefficients for Associations Between VRFs and FW Across White Matter Tracts**

**
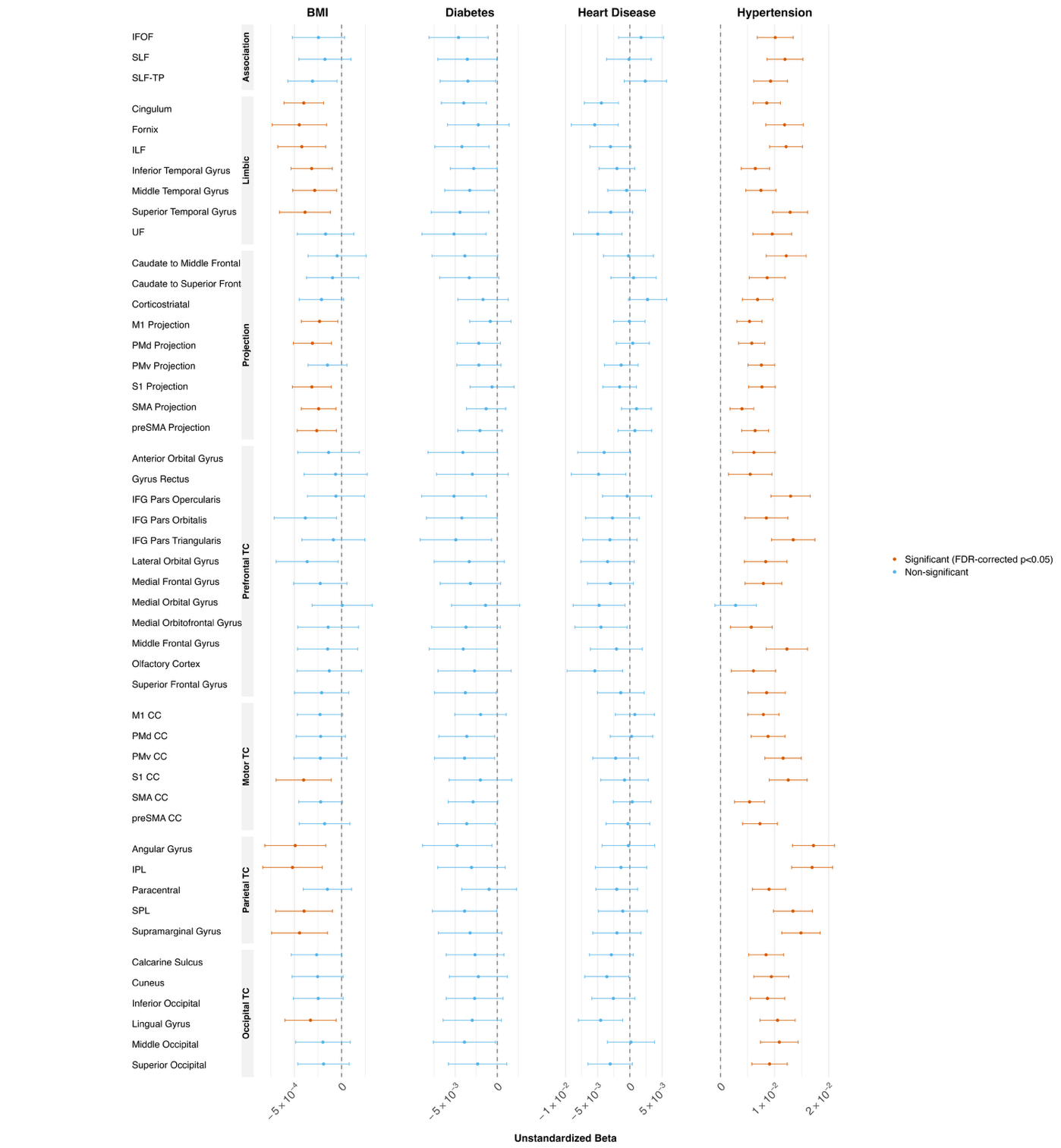
**

**Supplementary Figure 1 Caption.** The forest plots illustrate the beta coefficients and 95% confidence intervals to quantify the associations between VRFs and FW across the white matter tracts from the 5-year restricted primary analysis. Individual points represent the beta coefficient for each association and horizontal lines indicate the 95% confidence interval. Associations that statistically significant after FDR-adjustment are colored red. CC, corpus callosum; IFG, inferior frontal gyrus; IFOF, inferior fronto-occipital fasciculus; ILF, inferior longitudinal fasciculus; IPL, inferior parietal lobe; M1, primary motor cortex; PMd, dorsal premotor; PMv, ventral premotor; S1, primary somatosensory cortex; SLF, superior longitudinal fasciculus; SLF-TP, temporoparietal superior longitudinal fasciculus; SMA, supplementary motor area; SPL, superior parietal lobe; TC, transcallosal; UF, uncinate fascicu

**
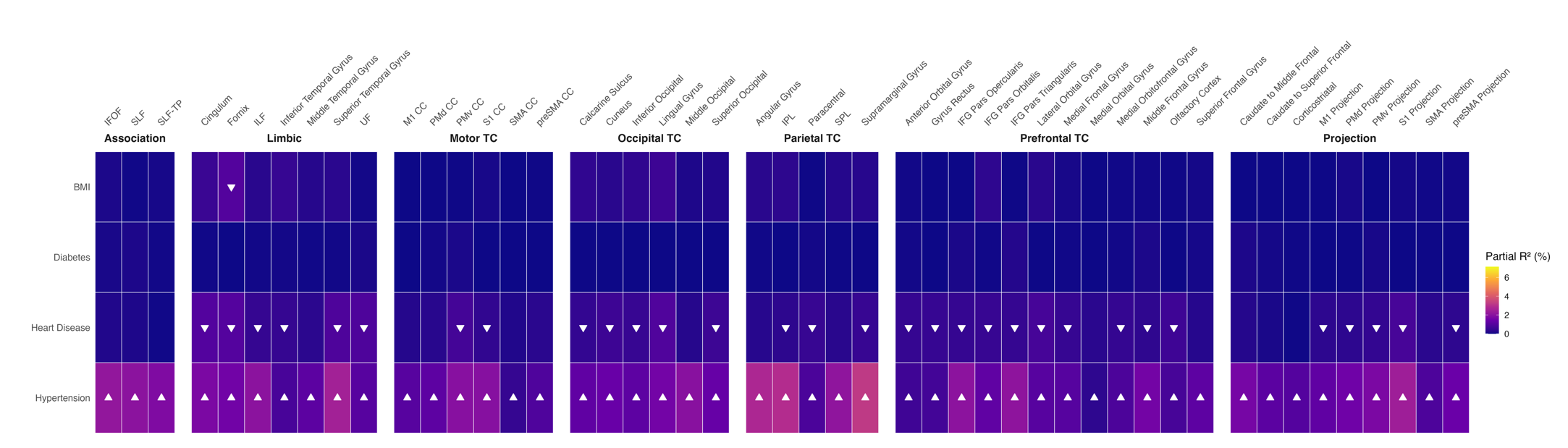
Supplementary Figure 2. Associations Between VRFs and FW from the 1-Year Restricted Analysis**

**Supplementary Figure 2 Caption.** Reduced models of the primary regression models were used to quantify the relative contributions of VRFs to alterations in FW across the 48 white matter tracts. The heatmap illustrates partial R^2^ values, highlighting the relative contributions to FW, from the 1-year restricted secondary analysis. *Tracts surviving correction for multiple comparisons. CC, corpus callosum; IFG, inferior frontal gyrus; IFOF, inferior fronto-occipital fasciculus; ILF, inferior longitudinal fasciculus; IPL, inferior parietal lobe; M1, primary motor cortex; PMd, dorsal premotor; PMv, ventral premotor; S1, primary somatosensory cortex; SLF, superior longitudinal fasciculus; SLF-TP, temporoparietal superior longitudinal fasciculus; SMA, supplementary motor area; SPL, superior parietal lobe; TC, transcallosal; UF, uncinate fasciculus.

**Supplementary Figure 3. Beta Coefficients for Associations Between VRFs and FW Across White Matter Tracts from the 1-Year Restricted Analysis**

**
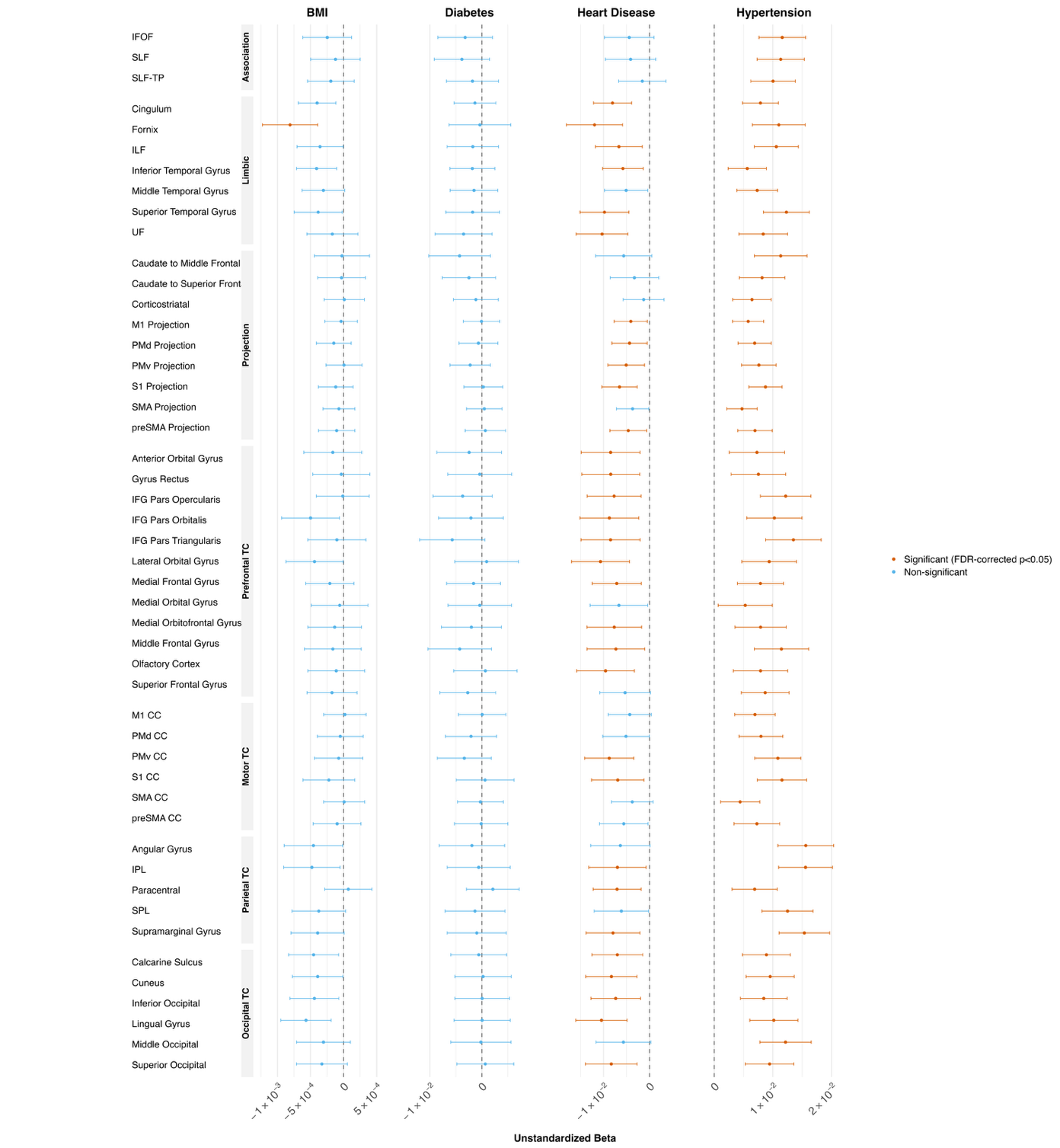
**

**Supplementary Figure 3 Caption.** The forest plots illustrate the beta coefficients and 95% confidence intervals to quantify the associations between VRFs and FW across the white matter tracts from the 1-year restricted secondary analysis. Individual points represent the beta coefficient for each association and horizontal lines indicate the 95% confidence interval. Associations that statistically significant after FDR-adjustment are colored red. CC, corpus callosum; IFG, inferior frontal gyrus; IFOF, inferior fronto-occipital fasciculus; ILF, inferior longitudinal fasciculus; IPL, inferior parietal lobe; M1, primary motor cortex; PMd, dorsal premotor; PMv, ventral premotor; S1, primary somatosensory cortex; SLF, superior longitudinal fasciculus; SLF-TP, temporoparietal superior longitudinal fasciculus; SMA, supplementary motor area; SPL, superior parietal lobe; TC, transcallosal; UF, uncinate fasciculus.

**Supplementary Figure 4. Beta Coefficients for Associations Between VRFs and FA_FWcorr_ Across White Matter Tracts**

**
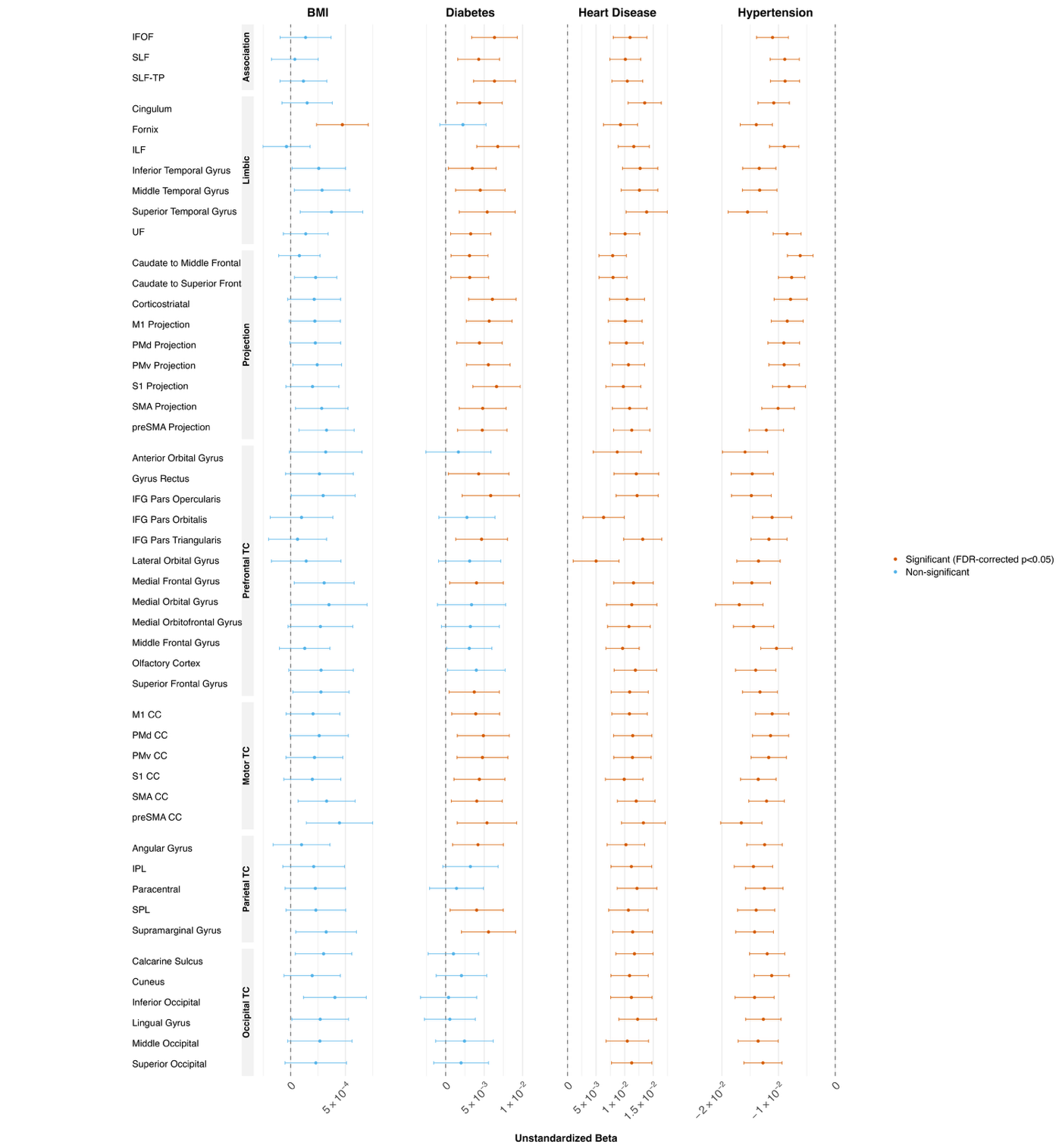
**

**Supplementary Figure 4 Caption.** The forest plots illustrate the beta coefficients and 95% confidence intervals to quantify the associations between VRFs and FA_FWcorr_ across the white matter tracts from the 5-year restricted primary analysis. Individual points represent the beta coefficient for each association and horizontal lines indicate the 95% confidence interval. Associations that statistically significant after FDR-adjustment are colored red. CC, corpus callosum; IFG, inferior frontal gyrus; IFOF, inferior fronto-occipital fasciculus; ILF, inferior longitudinal fasciculus; IPL, inferior parietal lobe; M1, primary motor cortex; PMd, dorsal premotor; PMv, ventral premotor; S1, primary somatosensory cortex; SLF, superior longitudinal fasciculus; SLF-TP, temporoparietal superior longitudinal fasciculus; SMA, supplementary motor area; SPL, superior parietal lobe; TC, transcallosal; UF, uncinate fasciculus.

**
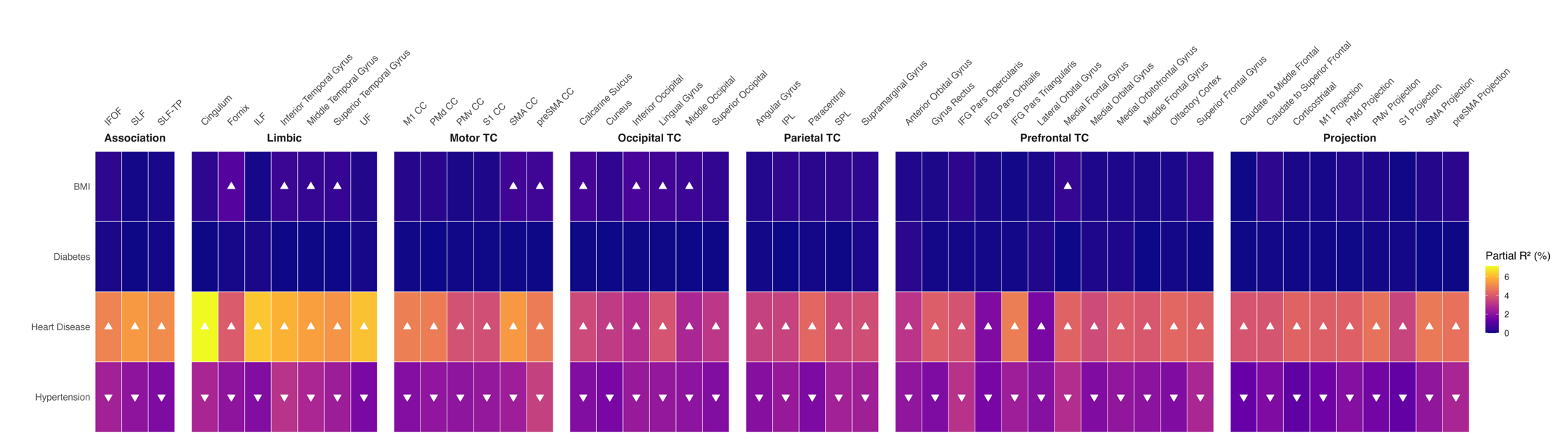
Supplementary Figure 5. Associations Between VRFs and FA_FWcorr_ from the 1-Year Restricted Analysis**

**Supplementary Figure 5 Caption.** Reduced models of the primary regression models were used to quantify the relative contributions of VRFs to alterations in FA_FWcorr_ across the 48 white matter tracts. The heatmap illustrates partial R^2^ values, highlighting the relative contributions to FA_FWcorr_, from the 1-year restricted secondary analysis. *Tracts surviving correction for multiple comparisons. CC, corpus callosum; IFG, inferior frontal gyrus; IFOF, inferior fronto-occipital fasciculus; ILF, inferior longitudinal fasciculus; IPL, inferior parietal lobe; M1, primary motor cortex; PMd, dorsal premotor; PMv, ventral premotor; S1, primary somatosensory cortex; SLF, superior longitudinal fasciculus; SLF-TP, temporoparietal superior longitudinal fasciculus; SMA, supplementary motor area; SPL, superior parietal lobe; TC, transcallosal; UF, uncinate fasciculus.

**Supplementary Figure 6. Beta Coefficients for Associations Between VRFs and FA_FWcorr_ Across White Matter Tracts from the 1-Year Restricted Analysis**

**
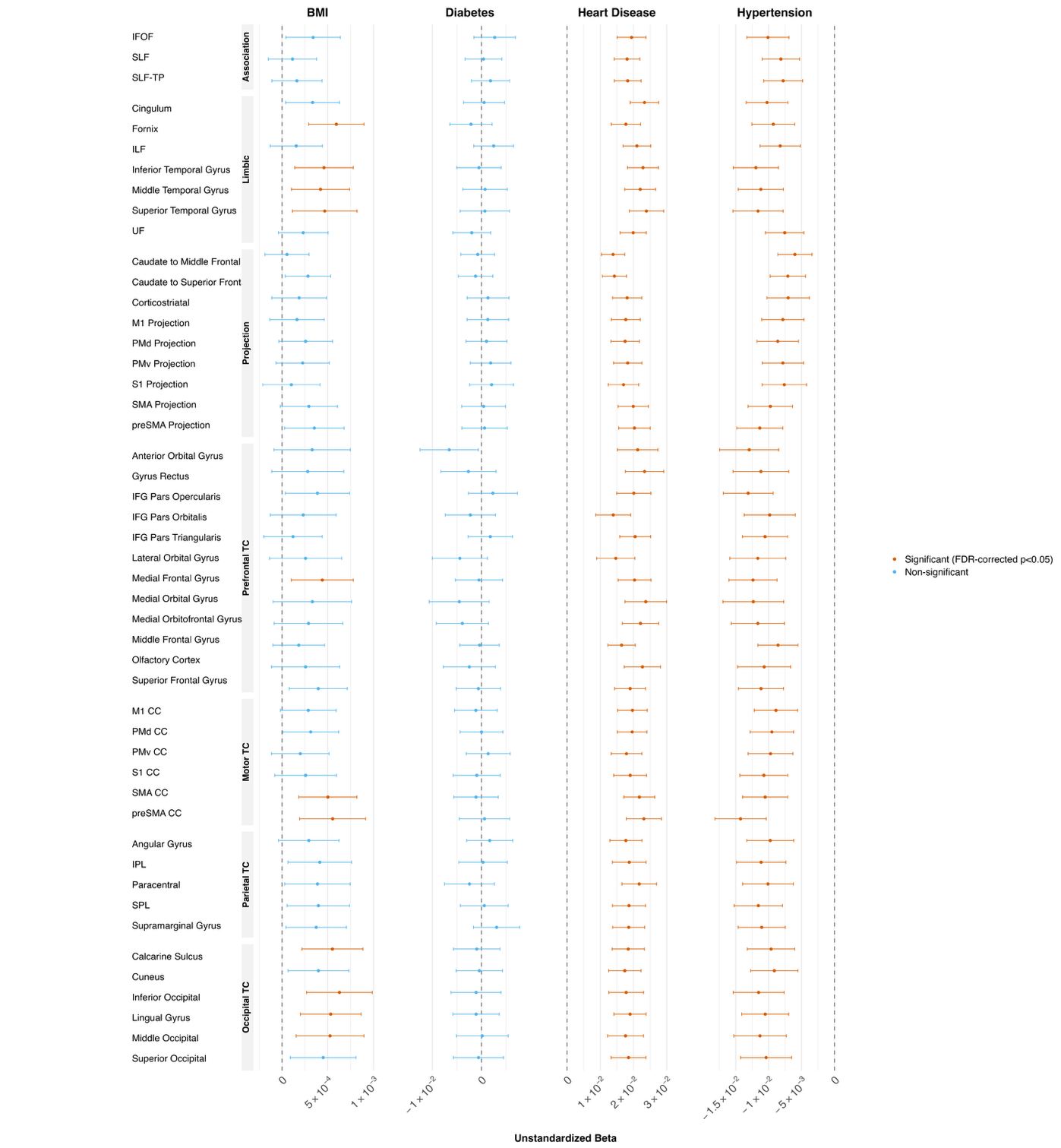
**

**Supplementary Figure 6 Caption.** The forest plots illustrate the beta coefficients and 95% confidence intervals to quantify the associations between VRFs and FA_FWcorr_ across the white matter tracts from the 1-year restricted secondary analysis. Individual points represent the beta coefficient for each association and horizontal lines indicate the 95% confidence interval. Associations that statistically significant after FDR-adjustment are colored red. CC, corpus callosum; IFG, inferior frontal gyrus; IFOF, inferior fronto-occipital fasciculus; ILF, inferior longitudinal fasciculus; IPL, inferior parietal lobe; M1, primary motor cortex; PMd, dorsal premotor; PMv, ventral premotor; S1, primary somatosensory cortex; SLF, superior longitudinal fasciculus; SLF-TP, temporoparietal superior longitudinal fasciculus; SMA, supplementary motor area; SPL, superior parietal lobe; TC, transcallosal; UF, uncinate fasciculus. **Supplementary Figure 7. Beta Coefficients for Associations Between VRFs and MD_FWcorr_ Across White Matter Tracts**

**
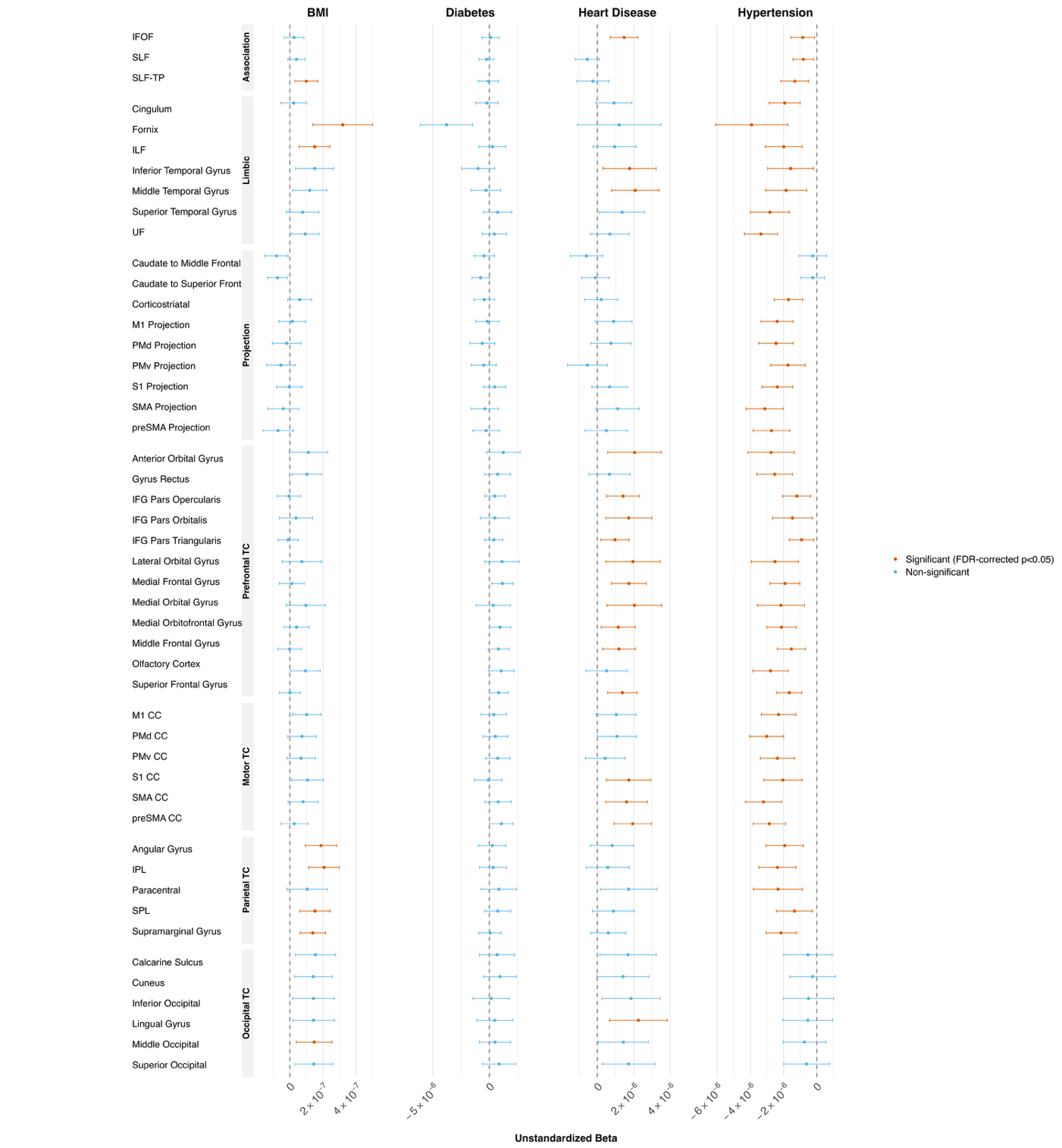
**

**Supplementary Figure 7 Caption.** The forest plots illustrate the beta coefficients and 95% confidence intervals to quantify the associations between VRFs and MD_FWcorr_ across the white matter tracts from the 5-year restricted primary analysis. Individual points represent the beta coefficient for each association and horizontal lines indicate the 95% confidence interval. Associations that statistically significant after FDR-adjustment are colored red. CC, corpus callosum; IFG, inferior frontal gyrus; IFOF, inferior fronto-occipital fasciculus; ILF, inferior longitudinal fasciculus; IPL, inferior parietal lobe; M1, primary motor cortex; PMd, dorsal premotor; PMv, ventral premotor; S1, primary somatosensory cortex; SLF, superior longitudinal fasciculus; SLF-TP, temporoparietal superior longitudinal fasciculus; SMA, supplementary motor area; SPL, superior parietal lobe; TC, transcallosal; UF, uncinate fasciculus.

**
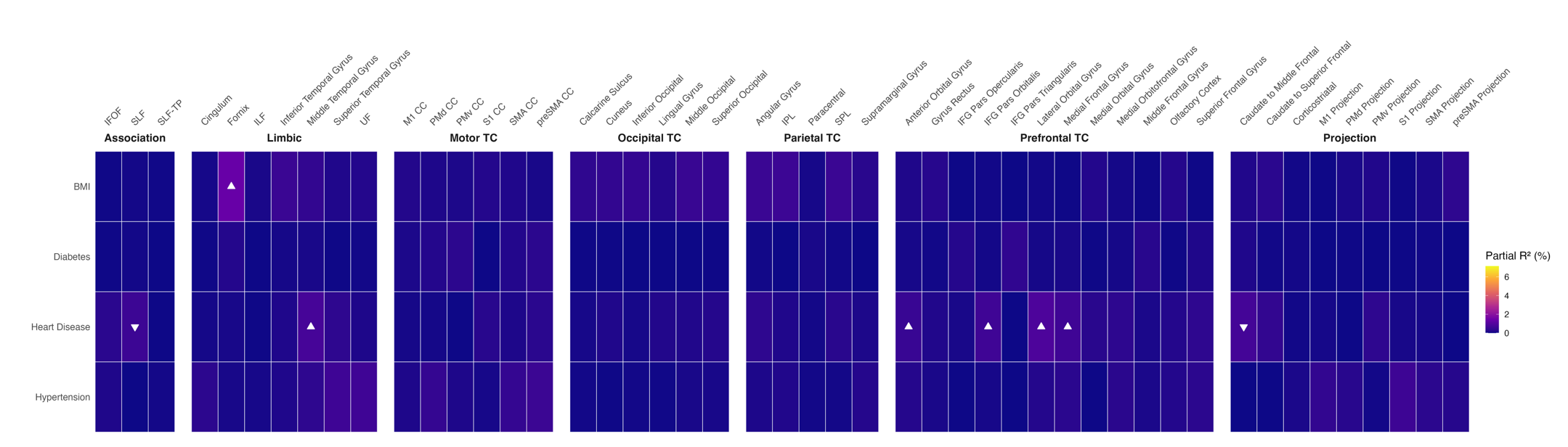
Supplementary Figure 8. Associations Between VRFs and MD_FWcorr_ from the 1-Year Restricted Analysis**

**Supplementary Figure 8 Caption.** Reduced models of the primary regression models were used to quantify the relative contributions of VRFs to alterations in MD_FWcorr_ across the 48 white matter tracts. The heatmap illustrates partial R^2^ values, highlighting the relative contributions to MD_FWcorr_, from the 1-year restricted secondary analysis. *Tracts surviving correction for multiple comparisons. CC, corpus callosum; IFG, inferior frontal gyrus; IFOF, inferior fronto-occipital fasciculus; ILF, inferior longitudinal fasciculus; IPL, inferior parietal lobe; M1, primary motor cortex; PMd, dorsal premotor; PMv, ventral premotor; S1, primary somatosensory cortex; SLF, superior longitudinal fasciculus; SLF-TP, temporoparietal superior longitudinal fasciculus; SMA, supplementary motor area; SPL, superior parietal lobe; TC, transcallosal; UF, uncinate fasciculus.

**Supplementary Figure 9. Beta Coefficients for Associations Between VRFs and MD_FWcorr_ Across White Matter Tracts from the 1-Year Restricted Analysis**

**
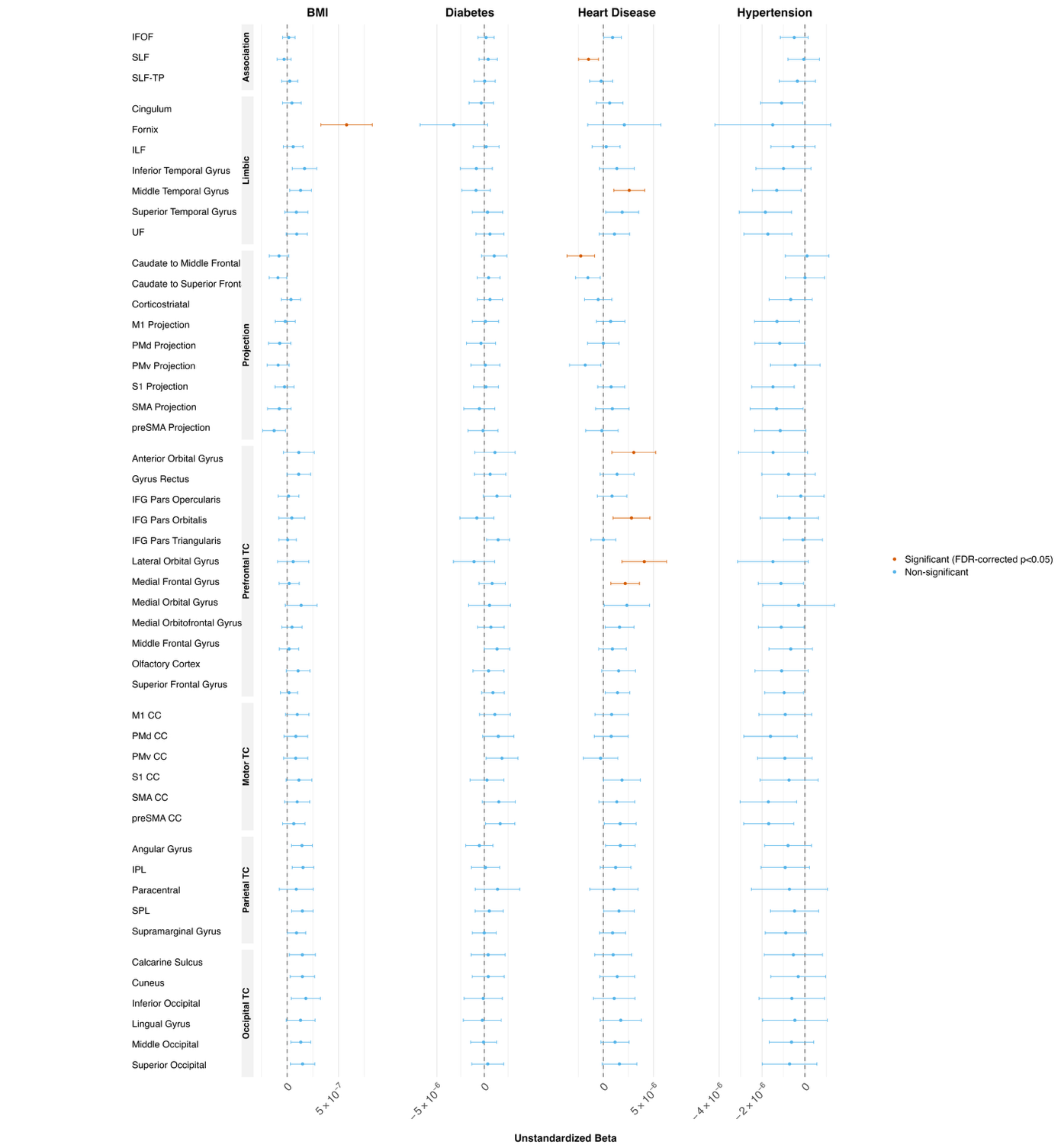
**

**Supplementary Figure 9 Caption.** The forest plots illustrate the beta coefficients and 95% confidence intervals to quantify the associations between VRFs and MD_FWcorr_ across the white matter tracts from the 1-year restricted secondary analysis. Individual points represent the beta coefficient for each association and horizontal lines indicate the 95% confidence interval. Associations that statistically significant after FDR-adjustment are colored red. CC, corpus callosum; IFG, inferior frontal gyrus; IFOF, inferior fronto-occipital fasciculus; ILF, inferior longitudinal fasciculus; IPL, inferior parietal lobe; M1, primary motor cortex; PMd, dorsal premotor; PMv, ventral premotor; S1, primary somatosensory cortex; SLF, superior longitudinal fasciculus; SLF-TP, temporoparietal superior longitudinal fasciculus; SMA, supplementary motor area; SPL, superior parietal lobe; TC, transcallosal; UF, uncinate fasciculus.

**Supplementary Figure 10. Beta Coefficients for Associations Between VRFs and AxD_FWcorr_ Across White Matter Tracts**

**
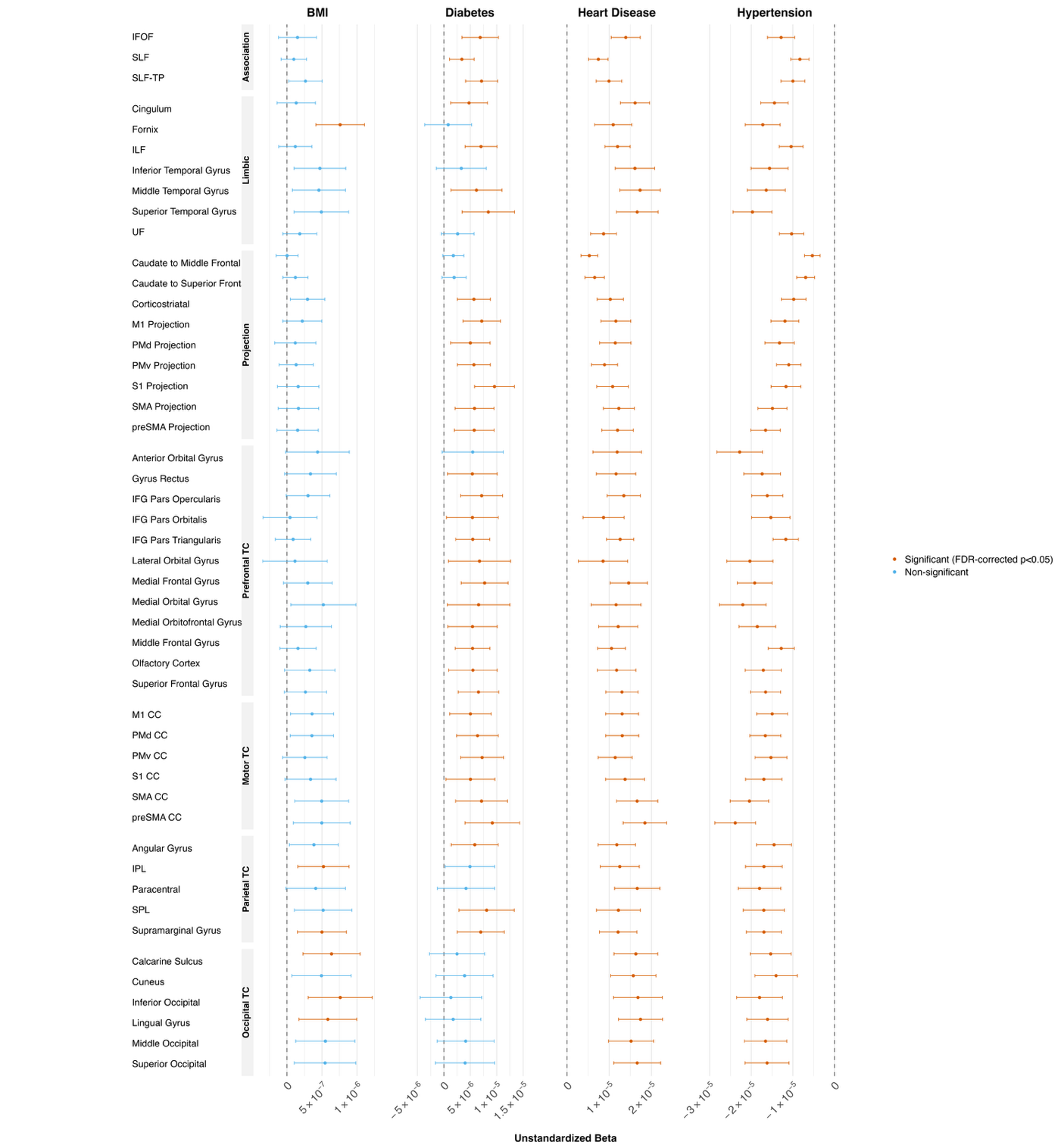
**

**Supplementary Figure 10 Caption.** The forest plots illustrate the beta coefficients and 95% confidence intervals to quantify the associations between VRFs and AxD_FWcorr_ across the white matter tracts from the 5-year restricted primary analysis. Individual points represent the beta coefficient for each association and horizontal lines indicate the 95% confidence interval. Associations that statistically significant after FDR-adjustment are colored red. CC, corpus callosum; IFG, inferior frontal gyrus; IFOF, inferior fronto-occipital fasciculus; ILF, inferior longitudinal fasciculus; IPL, inferior parietal lobe; M1, primary motor cortex; PMd, dorsal premotor; PMv, ventral premotor; S1, primary somatosensory cortex; SLF, superior longitudinal fasciculus; SLF-TP, temporoparietal superior longitudinal fasciculus; SMA, supplementary motor area; SPL, superior parietal lobe; TC, transcallosal; UF, uncinate fasciculus.

**Supplementary Figure 11. Associations Between VRFs and AxD_FWcorr_ from the 1-Year Restricted Analysis**

**
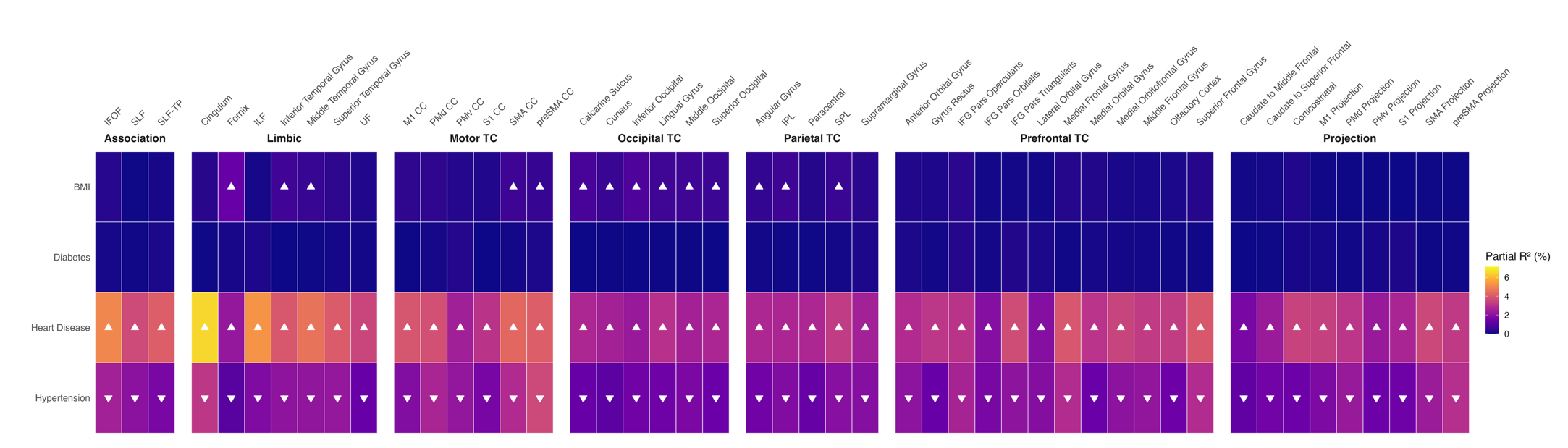
**

**Supplementary Figure 11 Caption.** Reduced models of the primary regression models were used to quantify the relative contributions of VRFs to alterations in AxD_FWcorr_ across the 48 white matter tracts. The heatmap illustrates partial R^2^ values, highlighting the relative contributions to AxD_FWcorr_, from the 1-year restricted secondary analysis. *Tracts surviving correction for multiple comparisons. CC, corpus callosum; IFG, inferior frontal gyrus; IFOF, inferior fronto-occipital fasciculus; ILF, inferior longitudinal fasciculus; IPL, inferior parietal lobe; M1, primary motor cortex; PMd, dorsal premotor; PMv, ventral premotor; S1, primary somatosensory cortex; SLF, superior longitudinal fasciculus; SLF-TP, temporoparietal superior longitudinal fasciculus; SMA, supplementary motor area; SPL, superior parietal lobe; TC, transcallosal; UF, uncinate fasciculus.

**Supplementary Figure 12. Beta Coefficients for Associations Between VRFs and AxD_FWcorr_ Across White Matter Tracts from the 1-Year Restricted Analysis**

**
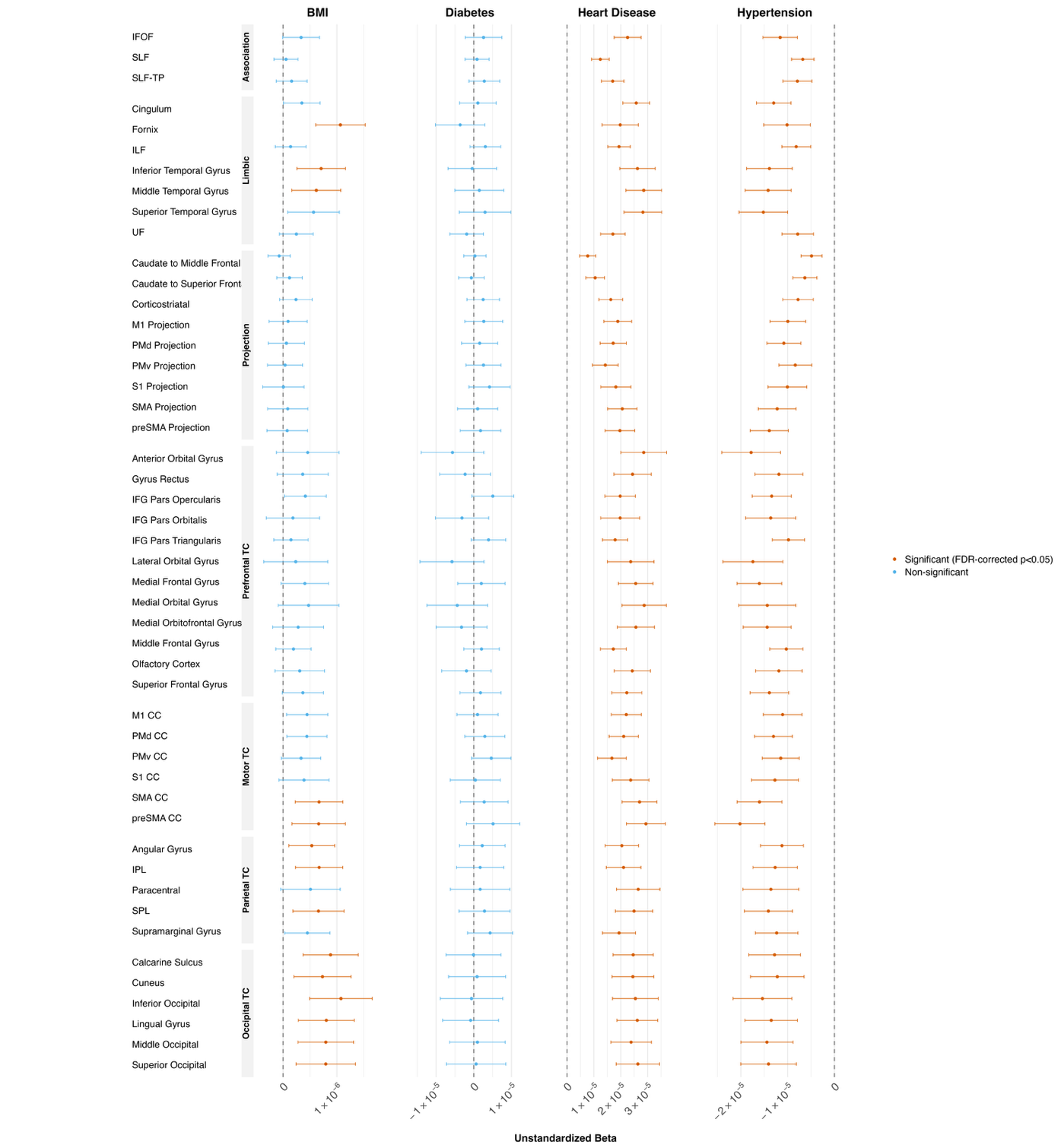
**

**Supplementary Figure 12 Caption.** The forest plots illustrate the beta coefficients and 95% confidence intervals to quantify the associations between VRFs and AxD_FWcorr_ across the white matter tracts from the 1-year restricted secondary analysis. Individual points represent the beta coefficient for each association and horizontal lines indicate the 95% confidence interval. Associations that statistically significant after FDR-adjustment are colored red. CC, corpus callosum; IFG, inferior frontal gyrus; IFOF, inferior fronto-occipital fasciculus; ILF, inferior longitudinal fasciculus; IPL, inferior parietal lobe; M1, primary motor cortex; PMd, dorsal premotor; PMv, ventral premotor; S1, primary somatosensory cortex; SLF, superior longitudinal fasciculus; SLF-TP, temporoparietal superior longitudinal fasciculus; SMA, supplementary motor area; SPL, superior parietal lobe; TC, transcallosal; UF, uncinate fasciculus.

**Supplementary Figure 13. Beta Coefficients for Associations Between VRFs and RD_FWcorr_ Across White Matter Tracts**

**
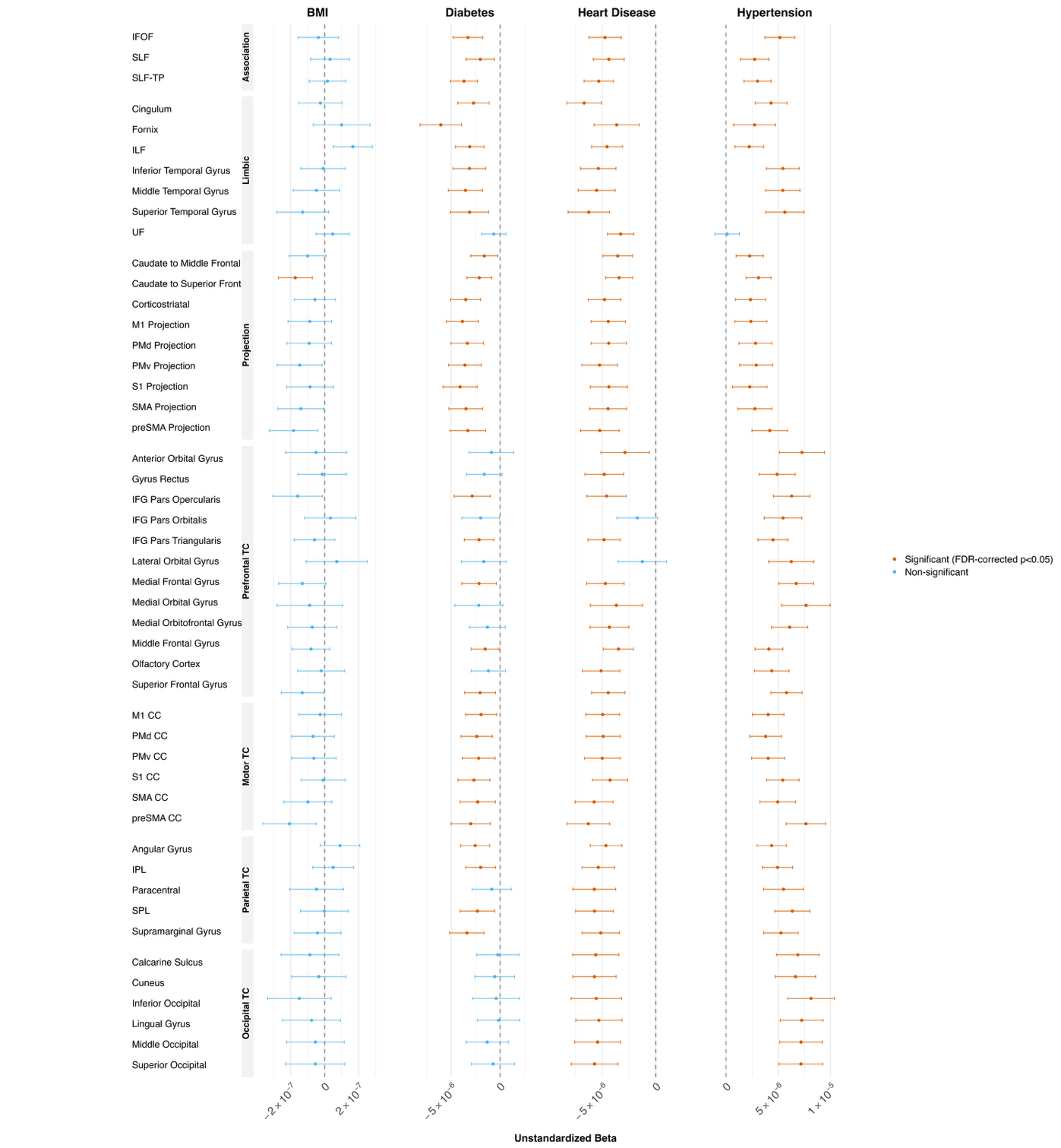
**

**Supplementary Figure 13 Caption.** The forest plots illustrate the beta coefficients and 95% confidence intervals to quantify the associations between VRFs and RD_FWcorr_ across the white matter tracts from the 5-year restricted primary analysis. Individual points represent the beta coefficient for each association and horizontal lines indicate the 95% confidence interval. Associations that statistically significant after FDR-adjustment are colored red. CC, corpus callosum; IFG, inferior frontal gyrus; IFOF, inferior fronto-occipital fasciculus; ILF, inferior longitudinal fasciculus; IPL, inferior parietal lobe; M1, primary motor cortex; PMd, dorsal premotor; PMv, ventral premotor; S1, primary somatosensory cortex; SLF, superior longitudinal fasciculus; SLF-TP, temporoparietal superior longitudinal fasciculus; SMA, supplementary motor area; SPL, superior parietal lobe; TC, transcallosal; UF, uncinate fasciculus.

**
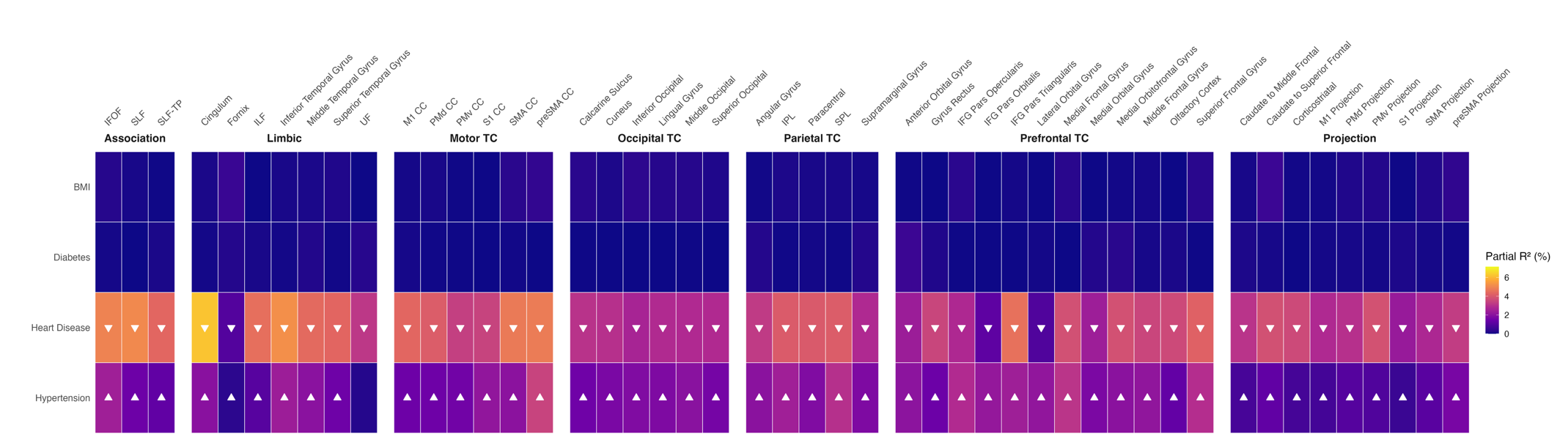
Supplementary Figure 14. Associations Between VRFs and RD_FWcorr_ from the 1-Year Restricted Analysis**

**Supplementary Figure 14 Caption.** Reduced models of the primary regression models were used to quantify the relative contributions of VRFs to alterations in RD_FWcorr_ across the 48 white matter tracts. The heatmap illustrates partial R^2^ values, highlighting the relative contributions to RD_FWcorr_, from the 1-year restricted primary analysis. *Tracts surviving correction for multiple comparisons. CC, corpus callosum; IFG, inferior frontal gyrus; IFOF, inferior fronto-occipital fasciculus; ILF, inferior longitudinal fasciculus; IPL, inferior parietal lobe; M1, primary motor cortex; PMd, dorsal premotor; PMv, ventral premotor; S1, primary somatosensory cortex; SLF, superior longitudinal fasciculus; SLF-TP, temporoparietal superior longitudinal fasciculus; SMA, supplementary motor area; SPL, superior parietal lobe; TC, transcallosal; UF, uncinate fasciculus.

**Supplementary Figure 15. Beta Coefficients for Associations Between VRFs and RD_FWcorr_ Across White Matter Tracts from the 1-Year Restricted Analysis**

**
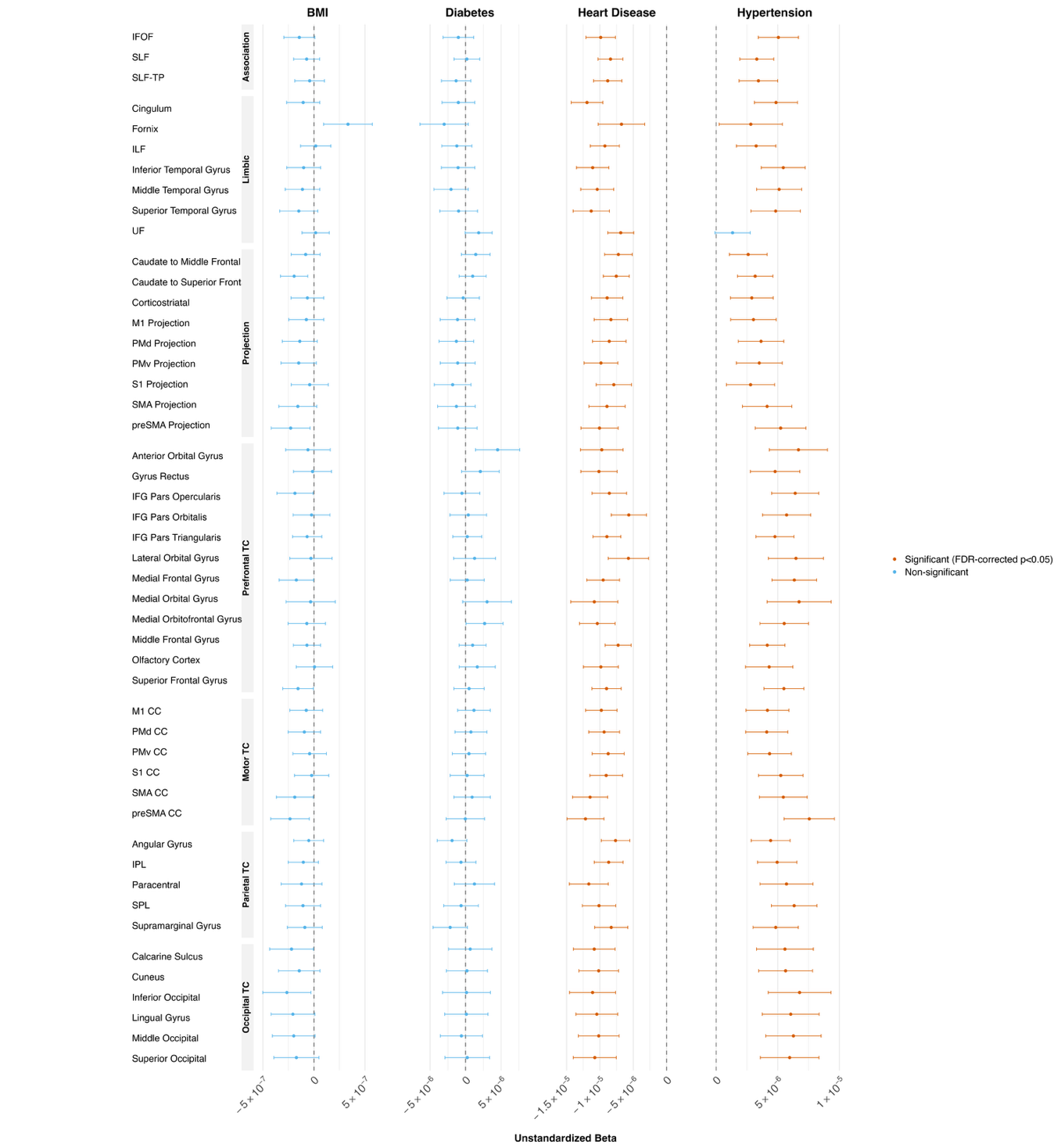
**

**Supplementary Figure 15 Caption.** The forest plots illustrate the beta coefficients and 95% confidence intervals to quantify the associations between VRFs and RD_FWcorr_ across the white matter tracts from the 1-year restricted secondary analysis. Individual points represent the beta coefficient for each association and horizontal lines indicate the 95% confidence interval. Associations that statistically significant after FDR-adjustment are colored red. CC, corpus callosum; IFG, inferior frontal gyrus; IFOF, inferior fronto-occipital fasciculus; ILF, inferior longitudinal fasciculus; IPL, inferior parietal lobe; M1, primary motor cortex; PMd, dorsal premotor; PMv, ventral premotor; S1, primary somatosensory cortex; SLF, superior longitudinal fasciculus; SLF-TP, temporoparietal superior longitudinal fasciculus; SMA, supplementary motor area; SPL, superior parietal lobe; TC, transcallosal; UF, uncinate fasciculus.
